# Clinical utility of family history of depression for prognosis of adolescent depression severity and duration assessed with predictive modeling

**DOI:** 10.1111/jcpp.13547

**Published:** 2021-11-30

**Authors:** Lisa S. Gorham, Neda Sadeghi, Lillian Eisner, Jeremy Taigman, Katherine Haynes, Karen Qi, Christopher C. Camp, Payton Fors, Diana Rodriguez, Jerry McGuire, Erin Garth, Chana Engel, Mollie Davis, Kenneth Towbin, Argyris Stringaris, Dylan M. Nielson

**Affiliations:** ^1^ Section of Clinical and Computational Psychiatry, Emotion and Development Branch National Institute of Mental Health National Institutes of Health Bethesda MD USA

**Keywords:** Depression, adolescence, family history, longitudinal studies

## Abstract

**Background:**

Family history of depression (FHD) is a known risk factor for the new onset of depression. However, it is unclear if FHD is clinically useful for prognosis in adolescents with current, ongoing, or past depression. This preregistered study uses a longitudinal, multi‐informant design to examine whether a child’s FHD adds information about future depressive episodes and depression severity applying state‐of‐the‐art predictive out‐of‐sample methodology.

**Methods:**

We examined data in adolescents with current or past depression (age 11–17 years) from the National Institute of Mental Health Characterization and Treatment of Adolescent Depression (CAT‐D) study. We asked whether a history of depression in a first‐degree relative was predictive of depressive episode duration (72 participants) and future depressive symptom severity in probands (129 participants, 1,439 total assessments).

**Results:**

Family history of depression, while statistically associated with time spent depressed, did not improve predictions of time spent depressed, nor did it improve models of change in depression severity measured by self‐ or parent‐report.

**Conclusions:**

Family history of depression does not improve the prediction of the course of depression in adolescents already diagnosed with depression. The difference between statistical association and predictive models highlights the importance of assessing predictive performance when evaluating questions of clinical utility.

## Introduction

Adolescent depression is a global public health problem. In 2017, 3.2 million U.S. adolescents experienced a depressive episode (NIMH, 2019). While it is well‐established that teens with a family history of depression (FHD) are at higher risk of developing depression, it is unclear whether FHD influences prognosis in currently depressed youth (Birmaher et al., 1996). Here, we use a longitudinal design to examine whether FHD is a clinically meaningful predictor of the duration or severity of future depressive episodes.

Family history of general psychopathology is a risk factor for developing depression (Kovacs, Devlin, & Pollock, 1997). Children of depressed parents are three times more likely to experience a lifetime episode of Major Depressive Disorder (MDD) (Birmaher et al., 1996). In adults, FHD is associated with earlier age of onset of MDD (Azorin, Belzeaux, Fakra, Hantouche, & Adida, 2016; Birmaher et al., 1996; Husain et al., 2009; Klein et al., 1999; Korten, Comijs, Lamers, & Penninx, 2012; Nierenberg et al., 2007; Tozzi et al., 2008) more chronic or recurrent depression (Hardeveld et al., 2013; Hölzel, Härter, Reese, & Kriston, 2011; Husain et al., 2009; van Loo, Aggen, Gardner, & Kendler, 2015, 2018; Patten et al., 2010), and more comorbid anxiety symptoms (Azorin et al., 2016; Husain et al., 2009). Furthermore, FHD and adverse life events may interact yielding an even greater risk for development of MDD (Monroe, Slavich, & Gotlib, 2014; Zimmermann et al., 2008).

However, the majority of studies examining the relationship between FHD and the course of depression are in adults. Very few have examined the relationship between FHD and clinical outcomes in currently depressed adolescents (Klein, Lewinsohn, Rohde, Seeley, & Durbin, 2002; Milne et al., 2009). Additionally, while studies have found that FHD is associated with earlier age of onset of depression, the upper age cut‐off defining ‘early age of onset’ ranges widely—as low as age 18 to as high as age 40 (Birmaher et al., 1996; Husain et al., 2009; Klein et al., 1999; Korten et al., 2012). By any of these definitions, adolescents with depression would have early onset and be at risk for a poorer prognosis. The question of whether the FHD adds anything to the prognosis for depression in teens is of clinical importance, since family history is relatively easy and inexpensive to obtain.

Previous studies have used FHD as an outcome itself, such as in Klein et al. (2002) and Milne et al. (2009). However, examining FHD as an outcome variable leaves unanswered whether FHD can be a useful predictor of prognosis among depressed adolescents. To address this, we followed depressed teenagers, ages 11 through 17, over time and hypothesized that: (a) FHD predicts the duration of depression (assessed using K‐SADS follow up clinical interviews) beyond what baseline symptom severity and adverse life events predict; and (b) FHD predicts future severity of depression as measured using the Mood and Feelings Questionnaire (MFQ). To test these hypotheses, we examined whether the addition of FHD to predictive models improved the fit of the models to the data in eightfold cross‐validation. This analysis was preregistered: https://osf.io/dt5v3?view_only=984f6a03087e439baab48c30052d08ac; data and code are here: https://github.com/lisagorham/Family_History_Project. Deviations from this preregistration are explained in the Supporting Information.

## Method

### Participants

Participants came from the NIMH Characterization and Treatment of Depression (CAT‐D) longitudinal study. Since 2017, this longitudinal study has tracked a cohort of teenagers who are healthy, have subthreshold depression (s‐MDD), or have a full diagnosis of depression. A description of recruitment procedures, inclusion/exclusion criteria, and visit frequency can be found in the Supporting Information. Additional inclusion criteria (Table 1) were used to create a subsample of this characterization cohort for the current analyses.

**Table 1:** Demographic characteristics of sample

Variable	Dataset for Question 1 (weeks of depression)	Dataset for Question 2 (depressive severity)
*N*	72	129
Number of assessments	144	1,439
Mean number of assessments	2	11.15 (*SD* = 6.71)
Interval between assessments (days)	Mean = 372.67 (*SD* = 25.19)	Median = 36.5 (IQR = 96)
Mean age (years)	15.83 (*SD* = 1.34)	15.61 (*SD* = 1.50)
% Female	72%	75%
% with a Positive FHD in a first degree relative	71%	74%
Mean MFQ Score	11.76 (*SD* = 6.93) (Collected at the Baseline Visit)	10.95 (*SD* = 5.32) (Across all people and timepoints)
% Taking an antidepressant at the baseline visit	47%	43%
% Taking another psychiatric medication at the baseline visit	32%	26%
Requirements for inclusion	MDD or s‐MDD participant	MDD or s‐MDD participant
	Family history interview complete	Family history interview complete
	Baseline MFQ score present	At least 2 MFQ scores present
	Value for weeks of depression collected at the 1 year follow up	
		Medication data from baseline
	Medication data from baseline + follow up	
	Value for CASE (stressful life events)	

### Family history interview

For this study, we modified an existing family history interview for parent(s)/guardian(s) focusing on disorders of interest and reducing time for administration demands (Merikangas, 2006). Ninety‐seven percent of participants completed interviews. We defined FHD by consolidating the family history data into a single index derived from having a first‐degree family member (biological parent or sibling) with a diagnosis of depressive disorder or symptoms of depressive disorder (Yes/No). Interview details and indices are described in the Supporting Information.

### Measures

Participant‐level clinical data were obtained from a combination of clinician assessments and self‐report questionnaires. Depressive symptom severity was measured by the short version of the Mood and Feelings Questionnaire (MFQ) (Angold, Costello, Messer, & Pickles, 1995; Thabrew, Stasiak, Bavin, Frampton, & Merry, 2018), which asks about symptoms occurring in the previous two weeks. MFQ was obtained at the baseline visit, at all annual follow up visits, and at multiple between visit intervals; most (88.37%) participants had several sequential measurements. Additionally, at the one‐year follow‐up visit, a master’s level clinician gathered the number of weeks of depression over the past year (the full follow up interval), using the Kiddy‐SADS‐PL DSM 5 depression screener and supplement. All cases were discussed in a case conference with two senior child psychiatrists present (KT and AS) where discrepancies were also resolved. To measure the construct of adverse life events at 1‐year follow‐up, we collected the Child and Adolescent Survey of Experiences (CASE) as a self‐rated questionnaire from each teen (Allen, Rapee, & Sandberg, 2012). Additional information about measures is available in the Supporting Information.

### Weeks of depression analyses

As preregistered, our first analysis examined the relationship between FHD and the number of weeks spent in a depressive episode from the baseline visit to the one‐year follow‐up. All models included covariates that are described in the table below. These were treated as nuisance variables to isolate the impact of FHD and depression severity on prediction. We also conducted an exploratory analysis using only variables that would have been available at baseline (see Supporting Information). In our analysis of weeks of depression, we fitted linear regressions for all models (Seabold & Perktold, 2010). The null model consisted of the variables described in the table below. We compared the out‐of‐sample predictive performance of this null model to four models of interest (Pedregosa et al., 2011). The first model of interest (FH) tested the additional predictive performance provided by FHD. The second model (MFQ) added baseline depression severity. The third model (MFQ+FH) used both FHD and baseline MFQ to test the incremental validity of FHD over baseline severity. The fourth model (MFQ+FH+CASE) includes the previous two variables plus stressful life events from the CASE and a test for the interaction between the CASE and FHD. To describe statistical associations, we first fitted each of these models to all of the data. Since these results are primarily descriptive, we did not correct p‐values for multiple comparisons.

We next assessed predictive performance with out‐of‐sample root mean squared error (RMSE) from eightfold cross‐validation with fold‐wise bootstrap confidence intervals. The RMSE is the quadratically weighted mean magnitude of errors; in other words, it gives a sense of the overall performance of a model and penalizes large errors more than small errors. We also provide the mean absolute error (MAE), which is the average magnitude of errors, to provide an intuitive sense of model performance. Finally, we assessed the significance of 11 model comparisons of interest (four here, seven in the Weeks of Depression Analyses portion of Appendix S1) with fold‐wise bootstrap. Exhaustive bootstrapping of eight folds yields 6,435 distinct combinations. We used one‐sided bootstrap tests to determine if adding FHD improved predictive performance of any model by more than 4 weeks. In this case, it is an upper‐tailed bootstrap, which was calculated as the proportion of bootstraps greater than the target value (4 weeks in this case). Plots were made with seaborn (Waskom, 2021) and Matplotlib (Hunter, 2007). In our plots we use a 99.9% confidence interval to account for the number of model comparisons (alpha of 0.05/11 models in main and Supporting Information = 0.0045, so an alpha of 0.001, corresponding to a 99.9% confidence interval, comfortably controls for multiple comparisons). Our threshold for a clinically meaningful difference in predictive performance was 2 weeks, the minimum length of a major depressive episode in DSM5 (American Psychiatric Association, 2013).

**Table:** 

Model	Formula
Null	Weeks of Depression ~ Antidepressants at Baseline + Other Meds at Baseline + Antidepressants at FU + Other Meds at FU + Inpatient + Sex + Age + Pandemic
FH	Weeks of Depression ~ FHD + Antidepressants at Baseline + Other Meds at Baseline + Antidepressants at FU + Other Meds at FU + Inpatient + Sex + Age + Pandemic
MFQ	Weeks of Depression ~ Baseline MFQ + Antidepressants at Baseline + Other Meds at Baseline + Antidepressants at FU + Other Meds at FU + Inpatient + Sex + Age + Pandemic
MFQ+FH	Weeks of Depression ~ Baseline MFQ + FHD + Antidepressants at Baseline + Other Meds at Baseline + Antidepressants at FU + Other Meds at FU + Inpatient + Sex + Age + Pandemic
MFQ+FH+CASE	Weeks of Depression ~ Baseline MFQ + FHD*SLEs + Antidepressants at Baseline + Other Meds at Baseline + Antidepressants at FU + Other Meds at FU + Inpatient + Sex + Age + Pandemic

### MFQ analyses

Our second question was if FHD was a significant predictor of future MFQ scores over and above current MFQ scores. Our methods yielded a variable number of MFQ scores for each participant (Figure S1), and so we conducted linear mixed effects modeling (Jolly, 2018). One participant was excluded from the analysis because of the large interval (900 days) between their assessments (the second largest visit interval was 518 days apart). All covariates are listed in the table below. We also included random effects of the time between scores predicted by each participant’s data, since each participant’s data collection was on a slightly different timeline. We compared the out‐of‐sample predictive performance of this null model to three primary models of interest (additional models described in the Supporting Information). As above, the first model of interest (FH) tested the additional predictive performance provided by FHD, the second model (MFQ) added current severity, and the third (MFQ+FHD) used both FHD and current MFQ to test the incremental validity of family history over current severity. We first fitted each of these models to all of the data in order to describe statistical associations. Since these results are primarily descriptive, we did not correct *p*‐values for multiple comparisons.

We next assessed predictive performance with out‐of‐sample RMSE from eightfold cross‐validation (Pedregosa et al., 2011). Finally, we assessed the significance of 12 model comparisons of interest (four here, eight in the Supporting Information) with fold‐wise bootstrap. We used one‐sided bootstrap tests to determine if adding FHD improved predictive performance of any model by more than 1 point on the MFQ, and to determine if any of the models have an RMSE >8 or <3. In plotting we use a difference in MFQ scores of six as an indicator of minimal clinical difference, the threshold for a meaningful difference on the child report short version of the MFQ (Liu & Adrian, 2019). Our plots use a 99.9% confidence interval to account for the number of model comparisons (alpha of 0.05/12 models in main and Supporting Information = 0.0042, so an alpha of 0.001, corresponding to a 99.9% confidence interval, comfortably controls for multiple comparisons).

**Table:** 

Model	Formula
Null Model	Next MFQ ~ Antidepressants at Baseline + Other Meds at Baseline + Interval + Inpatient + Previous Age + Sex + Pandemic + (1 + Interval | Participant ID)
FHD	Next MFQ ~ FHD*Interval + Antidepressants at Baseline + Other Meds at Baseline + Time Between the Two Scores + Inpatient + Previous Age + Sex + Pandemic + (1 + Interval | Participant ID)
MFQ	Next MFQ ~ Previous MFQ + Antidepressants at Baseline + Other Meds at Baseline + Inpatient + Previous Age + Sex + Pandemic + (1 + Interval | Participant ID)
MFQ + FHD	Next MFQ ~ Previous MFQ + FHD*Interval + Antidepressants at Baseline + Other Meds at Baseline + Inpatient + Previous Age + Sex + Pandemic + (1 + Interval | Participant ID)

## Results

Demographic characteristics of our sample are in Table 1. For our first set of analyses (FHD and weeks of depression), 71% of our probands had a FHD. For our second set of analyses (FHD and depressive symptom severity), 74% of our sample had a FHD. To further describe our sample, a simple χ^2^ test was performed to examine the likelihood of being depressed given a FHD. This was completed with our entire cohort of healthy volunteers and patients. Thirty‐four of 88 healthy volunteers had a FHD; 105 out of 140 depressed teenagers had a FHD (X^2^ = 28.516, *df* = 1, *p* < .001). Additionally, nine participants who started as healthy volunteers had onset of a depressive episode during the study; six of the nine had a FHD. Pairwise correlations for each model are described in the Supporting Information (Figures S2–S4).

### FHD and weeks of depression results

Detailed results from the linear regressions are in the Supporting Information (Tables S1–S5). Baseline MFQ score and FHD were both associated with weeks of depression, in addition to other medications at baseline (Table S3). When stressful life events (CASE) were added to the model as an interaction (Table S4), only baseline MFQ score and other medications at baseline were associated with weeks of depression.

**Figure 1:**
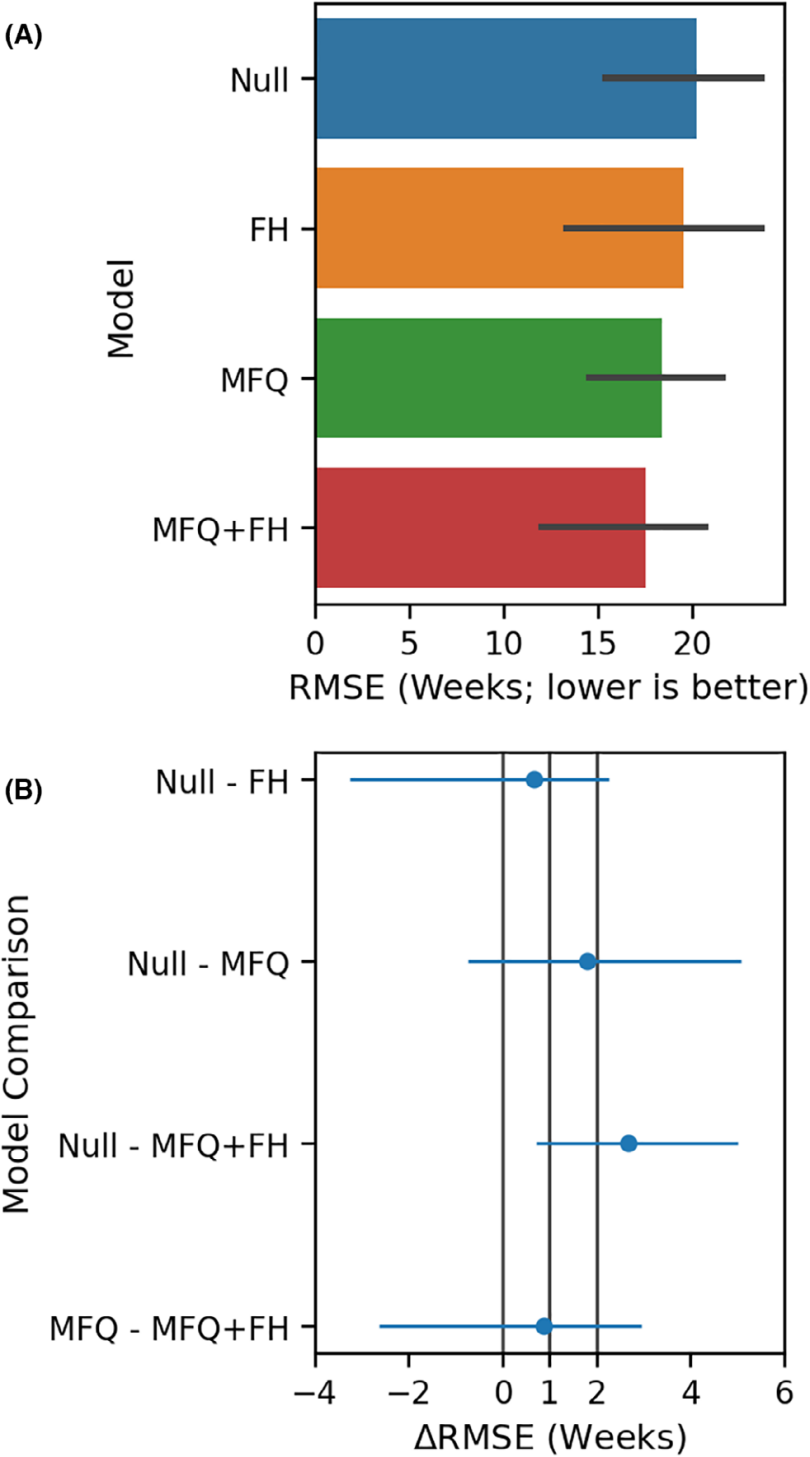
The top panel (A) shows the RMSE for each model along with bootstrap 99.9% confidence intervals. The lower panel (B) shows comparisons of interest between models in A. Each dot represents the mean difference in RMSE, while the error bars represent 99.9% confidence intervals. Two weeks of depression is the defined minimum length for an episode of depression, and half of this value is also shown. In no case does Family History improve the RMSE by more than 4 weeks (Null vs. MFQ, and MFQ vs. MFQ + FH)

We cross‐validated results to evaluate the predictive performance of each model (Figure 1A; Figure S5A). None of the models had a mean RMSE <10 weeks (one‐sided bootstrap *t*‐test, *p* = 9.32 × 10^−4^) and the addition of FHD to the Null and MFQ models did not improve RMSE by more than 4 weeks (one‐sided bootstrap *t*‐test, *p* = 1.6 × 10^−4^). The large uncertainties in this analysis are likely due to the relatively small sample size for this analysis (Supporting Information Results, Power Analysis for Weeks of Depression). We did not preregister analyses using MAE but present them here exploratorily as an additional performance metric. FHD did not improve the MAE of any model by more than 4 weeks (one sided bootstrap *t*‐test, *p* < 7.8 × 10^−4^), nor was there a difference in MAE between any tested pair of models greater than 6 weeks (one sided bootstrap *t*‐test, *p* = 1.6 × 10^−4^, Figure S5B). The CASE did not improve RMSE or MAE more than 2 weeks (one sided bootstrap t‐test, RMSE *p* = 1.6 × 10^−4^, MAE *p* = 1.6 × 10^−4^) when compared to a model including MFQ and FHD. These results indicate that while baseline MFQ score and FHD were associated with weeks of depression, neither they, nor the CASE, improve the average error in predicting weeks of depression by more than 6 weeks.

### FHD and future MFQ results

This analysis included 1,310 sequential pairs of assessments from 129 participants (Median ± IQR number of assessments = 10.0 ± 10.0; Median ± IQR Interval = 36.5 ± 96.0 days; Figure S6). When the previous MFQ score and FHD were used simultaneously to predict the next MFQ score, only the previous MFQ and male sex were associated with future depression severity (Table S6). When the model included the baseline MFQ, previous MFQ, and FHD, baseline MFQ score, previous MFQ score, and male sex were associated with the next MFQ score, but FHD was not (Table S7). Complete results for all models can be found in Tables S6–S14.

**Figure 2:**
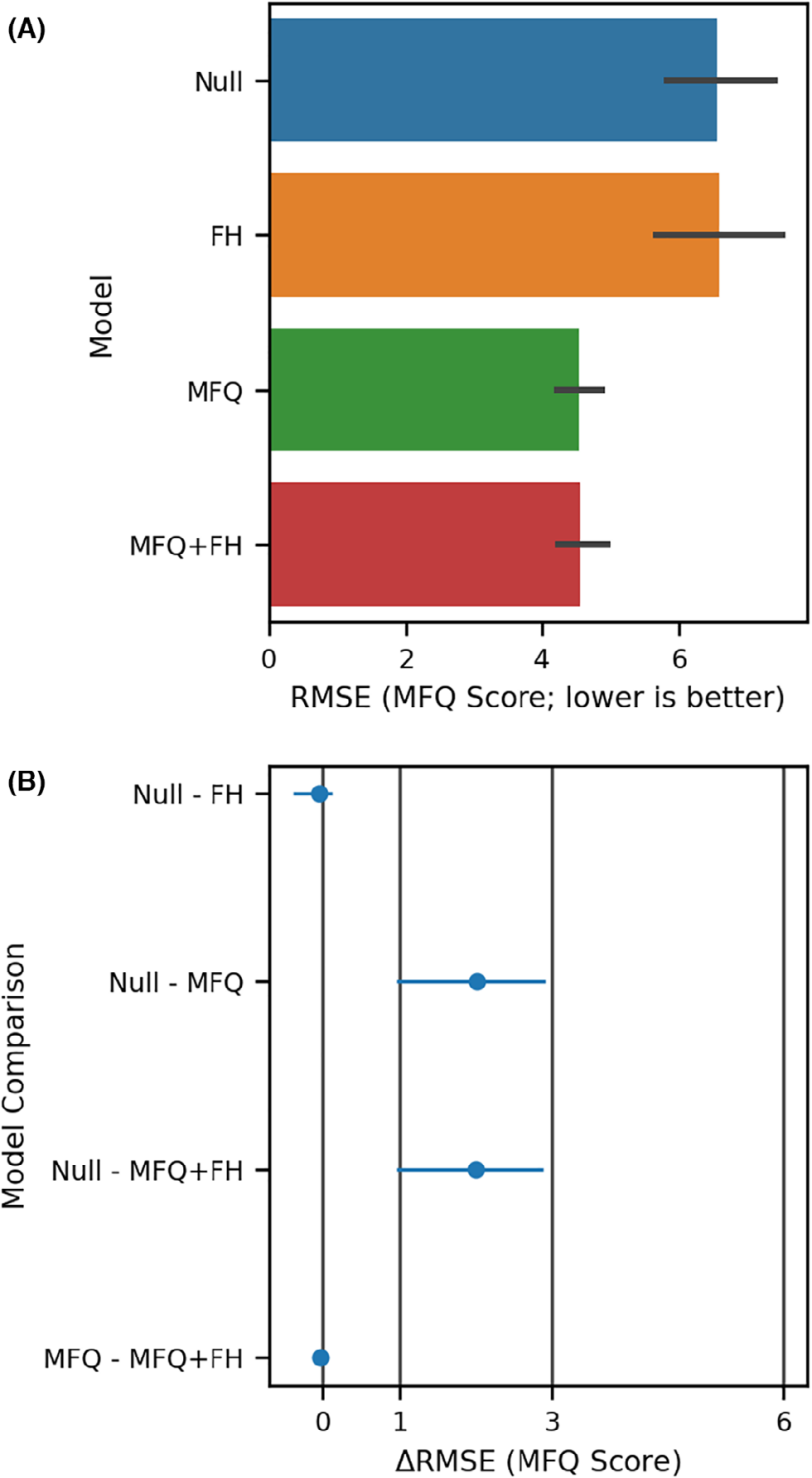
The top panel (A) shows the RMSE for each model along with bootstrap 99.9% confidence intervals. The lower panel (B) shows comparisons of interest between models in A. Each dot represents the mean difference in RMSE, while the error bars represent 99.9% confidence intervals. Six points on the MFQ is the minimum clinical difference, and half of this value is also shown

In cross‐validated results evaluating the predictive performance of each model (Figure 2A; Figure S7A), all of the models (including the Null) had an RMSE between 3 and 8 points on the MFQ (two one‐sided bootstrap *t*‐tests, *p* < 1.6 × 10^−4^). FHD did not improve the performance of any model by more than 1 point (one sided bootstrap *t*‐test, *p* < 1.6 × 10^−4^, Figure 2B; Figure S7B). We also tested a quadratic model as preregistered, but it did not change the RMSE of any model by more than one point (one sided bootstrap *t*‐test, *p* < 3.2 × 10^−4^, Figure S7B). In an exploratory analysis, results using MAE were similar to those with RMSE (Figure S7). To summarize, we did not find that FHD was associated with subsequent depression severity, and including FHD in predictive models of depression severity did not improve those models in a clinically meaningful way.

### Sensitivity analyses

We conducted several additional exploratory analyses to verify that our findings were robust. All of these analyses, which are described in detail in the Supporting Information, produced similar results to the primary analyses. In particular: (a) we repeated the analyses for weeks of depression using only the variables that would have been available at the baseline visit (Supporting Information, Tables S15–S19); (b) we repeated the analyses with FHD restricted to only family members with a formal diagnosis of depression (Figures S8 and S9); (c) we also considered a first degree relative with a formal diagnosis or symptoms of anxiety instead of depression (Figures S10 and S11); (d) we originally included participants who spent time in our inpatient unit, so we repeated the analyses with these participants excluded (Figures S12 and S13); (e) we repeated the analyses with elastic net regression and randomized trees regression instead of linear models and the results concurred with our previous findings (Figure S14); (f) we used parent‐report MFQ instead of self‐report MFQ (Table S20, Figure S15); (g) we analyzed subsets of the MFQ data with short (1–15 days), medium (16–77 days), and long (77–518 days) intervisit intervals. We also examined prediction of the final MFQ score from the first MFQ score, which gave a median intervisit interval of 727 days (Table S21, Figure S16).

We also confirmed our results with alternative analytical approaches (Figure S17). We explored alternative mixed effects models for evidence of a relationship between FHD and depression trajectories and found no such evidence (Appendix S1, Sensitivity Analyses). Finally, we conducted a linear discriminant analysis to see if patterns of depressive symptoms at baseline differed based on the presence of FHD—there was no significant difference in symptom patterns (Appendix S1, Sensitivity Analyses).

## Discussion

We examined whether, in a cohort of teens with past or current depression, FHD in a first‐degree relative is a clinically useful predictor of depressive episode duration and future severity. We hypothesized that FHD would predict the duration of time spent depressed and the severity of depression at the baseline visit. We found that, while FHD was associated with duration, it was of no additional predictive value, reducing error by less than a week. Next, we hypothesized that FHD would interact with stressful life events to predict depressive episode duration. However, we could not conclude if this combined model improved prediction compared to null models on the basis of RMSE or MAE, due to large variability. Finally, we hypothesized that FHD would predict future depressive severity beyond that predicted by current symptom severity, but it did not reduce prediction error by more than one point on the MFQ.

In this study, we examined cross‐validated predictive accuracy. The significance of regressors in a linear model may be a result of overfitting and is not necessarily indicative of the usefulness of those regressors in clinical practice (Poldrack, Huckins, & Varoquaux, 2020). By looking at changes in predictive performance when adding FHD and CASE, we can get a better estimate of the likelihood that these measures will be generally informative. Comparing the changes in predictive performance to established cut‐offs of clinical significance allows us to investigate the possibility that they will provide meaningful information for clinicians. In this case, we found it unlikely that FHD will be clinically informative for predicting the time someone is likely to spend depressed, or the severity of their depression at a subsequent visit.

This could be due to several reasons. First, previous studies, using multivariate analyses, reported that FHD is a significant predictor of chronicity/recurrence. However, they did not assess whether FHD adds additional predictive value beyond other variables (ex. van Loo, Aggen, Gardner, & Kendler, 2018). Moreover, other studies applied dissimilar definitions or measures of chronic or recurrent depression. For example, Hardeveld et al. (2013) used the time to recurrence, while Milne et al. (2009) used the number of times that a depressive episode was diagnosed. Others provide no definition and imply that recurrence is anything more than a single depressive episode (Klein et al., 2002). Another reason why our results diverge from those of others is that ours is the first study, to our knowledge, to explicitly look at the relationship between FHD and the number of weeks of depression in a sample of depressed adolescents. Studies in other populations produced mixed results. Kendler, Neale, Kessler, Heath, and Eaves (1994) reported the duration of the longest depressive episode is a poor predictor of MDD in a cotwin, while Patten et al. (2010) found the opposite (an odds ratio of 1.5 in predicting weeks of depression) and Husain et al. (2009) reported that those with a FHD had a longer duration of illness.

In contrast to other studies, we found that FHD did not interact with stressful life events to predict depressive episode duration. Others report a relationship between FHD, stressful life events, and depression (Luby, Belden, & Spitznagel, 2006; Monroe et al., 2014; Zimmermann et al., 2008). We collected information on stressful life events only at the one‐year follow‐up and thus were unable to assess the role of such events at baseline. Additionally, other studies did not use the outcome variable of duration of depressive episode. Zimmermann et al. (2008) and Monroe et al. (2014) looked at incidence of depression, and Luby et al. (2006) examined depressive episode severity, rather than duration. While both severity and duration are important, they answer different clinical questions and provide different information to clinicians looking for indicators of prognosis.

Finally, we found that FHD did not improve the prediction of future depressive symptom severity in our adolescents, using either self‐report or parent‐report measures. In our study, we quantified depressive symptom severity using the MFQ, which we selected because it was developed specifically for adolescents with depression. FHD was associated with depression severity in preschoolers (Luby et al., 2006) and adults (Janzing et al., 2009), although not in other studies (Husain et al., 2009; Nierenberg et al., 2007; Richards et al., 2016) or only as a trend relationship (Milne et al., 2009). Such differences could be produced by applying different definitions of impairment/severity or using different scales (e.g. HAM‐D, QIDS‐C, BDI, more broad designations, or impairment ratings on a scale from 1 to 5). Our study was the first, to our knowledge, to look at the relationship between FHD and score on the MFQ. While it is possible that FHD does not predict future depressive severity, another possibility is that FHD does not predict severity as measured by the MFQ; other quantifications of episode severity could exhibit stronger associations.

### Limitations

There were a number of limitations to our study. First, whilst our analyses for change in depression scores (as measured using the MFQs) are very well powered, providing 80% power to detect change of less than one point for the MFQ (Supporting Information, Power Analysis for MFQ; Figure S18), our sample was relatively small for the weeks of depression analysis. The increased power to detect differences with the MFQ analyses is due to the availability of repeated measures and therefore observations; by contrast, weeks of depression is a singular measure. We addressed this weakness by using one‐sided tests and established thresholds for clinical significance to determine that it is unlikely that FHD will be clinically informative for weeks of depression or depression severity. Based on simulations and power analyses, our cross‐validated analysis of weeks of depression was only sufficiently powered to detect a difference of 10 weeks RMSE (Supporting Information Results, Power Analysis for Weeks of Depression; Figure S19). The two‐week clinical significance threshold we selected is quite stringent. Even if we had not used a cross‐validated approach, we would have needed 246 participants in order to have 80% power to detect a change in model fit of this size. Second, over 70% of our participants were female. FHD could have a different effect in males than females that we were unable to detect. Third, data on family members were obtained from a sole informant (typically one parent). This method risks collecting incorrect or incomplete information about psychiatric histories of family members. Family history studies typically interview every member in the family to improve the reliability and validity of the data. This was not possible with our sample. Additionally, our sample is not representative of the national population of adolescents, nor is it necessarily representative of all adolescents with depression. Also, our family history interview captured data on other disorders (e.g. mania, schizophrenia, autism, eating disorders, and anxiety) and it may be useful to investigate whether a family history of these other disorders affects clinical outcomes. Finally, it would be helpful to investigate whether FHD relates to single‐episode versus recurrent depression (Monroe, Anderson, & Harkness, 2019), provides insight into subtypes of depressive symptoms, has an impact over longer time periods, or is particularly relevant for predicting treatment response in medication‐naive adolescents.

## Conclusion

FHD was associated with the number of weeks that an adolescent spent depressed over the course of a year, but it did not add predictive value. We also found that FHD did not predict future depressive symptom severity beyond that predicted by current symptom severity. In this sample of depressed adolescents, FHD may be a better indicator of broader variables such as depressive incidence or age of onset, rather than the narrower clinical variables of time spent depressed or future severity.

## Supporting information


**Appendix S1**. Supplementary Methods and Results.
**Table S1**. Linear regression results for null model.
**Table S2**. Linear regression results for MFQ model.
**Table S3**. Linear regression results for MFQ + FH model.
**Table S4**. Linear regression results for MFQ + FH + CASE model.
**Table S5**. Linear regression results for FH model.
**Table S6**. Linear mixed effect results for Model 2 (Null Model + Previous MFQ + Family History).
**Table S7**. Linear mixed effect results for MFQ0 + MFQ + FH model.
**Table S8**. Linear mixed effect results for Null model.
**Table S9**. Linear mixed effect results for Model 1 (Null Model + Previous MFQ).
**Table S10**. Linear mixed effect results for Model 3 (Null Model + Family History).
**Table S11**. Linear mixed effect results for Model 4 (Quadratic Family History Model).
**Table S12**. Linear mixed effect results for MFQ0 model.
**Table S13**. Linear mixed effects results for MFQ0 + FH model.
**Table S14**. Linear mixed effect results for MFQ0 + MFQ model.
**Table S15**. Demographic characteristics of sample.
**Table S16**. Linear regression results for baseline Null model.
**Table S17**. Linear regression results for baseline FH model.
**Table S18**. Linear regression results for baseline MFQ model.
**Table S19**. Linear regression results for baseline MFQ + FH model.
**Table S20**. Demographic characteristics of sample for parent report sensitivity analysis.
**Table S21**. Demographic characteristics of sample for sensitivity analysis of different intervisit interval subsets for the analysis of depressive severity.
**Figure S1**. Spaghetti plots of all of the MFQ trajectories.
**Figure S2**. Pairwise Pearson correlations between terms in the Weeks of Depression analysis.
**Figure S3**. Pairwise Pearson correlations between terms with a single value per participant in the MFQ analysis.
**Figure S4**. Pairwise Pearson correlations between terms with a unique value per pair of visits in the MFQ analysis.
**Figure S5**. These are the expanded results for prediction of weeks of depression.
**Figure S6**. Distributions of data included in MFQ analysis.
**Figure S7**. These are the expanded results for prediction of depression severity as measured by the MFQ.
**Figure S8**. This shows the sensitivity analysis using family history of depression diagnosis (as opposed to “some symptoms” and/or diagnosis) as a predictor of weeks of depression.
**Figure S9**. This shows the sensitivity analysis using family history of depression diagnosis (as opposed to “some symptoms” and/or diagnosis) as a predictor of depression severity as measured by MFQ.
**Figure S10**. This shows the sensitivity analysis using family history of anxiety (FHA) as a predictor of weeks of depression.
**Figure S11**. This shows the sensitivity analysis using family history of anxiety (FHA) as a predictor of depression severity as measured by MFQ.
**Figure S12**. This shows the sensitivity analysis excluding all current and former inpatients with family history of depression as a predictor of weeks of depression.
**Figure S13**. This shows the sensitivity analysis excluding all current and former inpatients with family history of depression as a predictor of depression severity as measured by the MFQ.
**Figure S14**. Comparison of changes in model performance when using Linear Models, Elastic Net, and Extra Trees.
**Figure S15**. This shows the sensitivity analysis with family history of depression as a predictor of depression severity as measured by parent‐report MFQ.
**Figure S16**. Comparison of differences in model performance for predicting depression severity as measured by the MFQ from family history of depression when using visits separated by different intervals.
**Figure S17**. The top panel (A) shows the unweighted root mean squared error (RMSE) and mean absolute error (MAE) for each model along with bootstrap 99.9% confidence intervals.
**Figure S18**. Power curves for different sample sizes for the MFQ analysis.
**Figure S19**. Power curves for different sample sizes for the Weeks of Depression analysis.
