# Supplementary material for: Clinical utility of family history of depression for prognosis of adolescent depression severity and duration assessed with predictive modeling
Source: J Child Psychol Psychiatry. 2021 Nov 30;63(8):939–47. doi: 10.1111/jcpp.13547 (PMC9541414; doi:10.1111/jcpp.13547)
Supplement: Supplementary file 1 — Appendix S1. Supplementary Methods and Results. Table S1. Linear regression results for null model. Table S2. Linear regression results for MFQ model. Table S3. Linear regression results for MFQ + FH model. Table S4. Linear regression results for MFQ + FH + CASE model. Table S5. Linear regression results for FH model. Table S6. Linear mixed effect results for Model 2 (Null Model + Previous MFQ + Family History). Table S7. Linear mixed effect results for MFQ0 + MFQ + FH model. Table S8. Linear mixed effect results for Null model. Table S9. Linear mixed effect results for Model 1 (Null Model + Previous MFQ). Table S10. Linear mixed effect results for Model 3 (Null Model + Family History). Table S11. Linear mixed effect results for Model 4 (Quadratic Family History Model). Table S12. Linear mixed effect results for MFQ0 model. Table S13. Linear mixed effects results for MFQ0 + FH model. Table S14. Linear mixed effect results for MFQ0 + MFQ model. Table S15. Demographic characteristics of sample. Table S16. Linear regression results for baseline Null model. Table S17. Linear regression results for baseline FH model. Table S18. Linear regression results for baseline MFQ model. Table S19. Linear regression results for baseline MFQ + FH model. Table S20. Demographic characteristics of sample for parent report sensitivity analysis. Table S21. Demographic characteristics of sample for sensitivity analysis of different intervisit interval subsets for the analysis of depressive severity. Figure S1. Spaghetti plots of all of the MFQ trajectories. Figure S2. Pairwise Pearson correlations between terms in the Weeks of Depression analysis. Figure S3. Pairwise Pearson correlations between terms with a single value per participant in the MFQ analysis. Figure S4. Pairwise Pearson correlations between terms with a unique value per pair of visits in the MFQ analysis. Figure S5. These are the expanded results for prediction of weeks of depression. Figure S6. Distributions of data includ [file JCPP-63-939-s001.docx]

**Supporting Information**

**Appendix S1**

**Characterization and Treatment of Depression (CAT-D) Longitudinal Adolescent Sample**

Participants came from the NIMH Characterization and Treatment of Depression (CAT-D) longitudinal study. This cohort tracks a number of healthy, subthreshold depression, and depressed teenagers over the course of many years. Participants are recruited locally and nationwide via mailings to selected physicians, announcements in newsletters, and contacts with support groups and approved websites. All participants are paid for their participation.

To be included as an MDD participant in this characterization study, one must be between the ages of 11-17 at the time of enrolment and must have a current diagnosis of MDD via the DSM-5 within the last six months. To be considered as an inpatient, they also must be failing their treatment as defined as a current CGAS score of less than 60 and must have a treater that believes it is clinically appropriate to change the child’s current treatment. Additionally, the research team must agree that the child’s response to their current treatment is no more than minimal.

To be included as an s-MDD (subthreshold depression) participant in this characterization study, one must be between the ages of 11-17 at the time of enrolment, must have an episode of depressed mood or loss of interest or pleasure lasting at least one week, and must have at least two of the seven other DSM-5 associated symptoms for major depression occurring in the last six months.

Finally, to be included as a healthy volunteer (HV) participant in this characterization study, one must be between the ages of 11-17 at the time of enrolment, must be competent to assent (and parents must be competent to consent), must be willing to participate in research and be willing to undergo psychiatric interviews, must speak English, and must have a primary care clinician in the community.

Exclusionary criteria for all participants include: a diagnosis of schizophrenia, schizophreniform disorder, schizoaffective illness, bipolar disorder, severe Autism Spectrum Disorder, Anorexia Nervosa, or other severe eating disorders; an IQ of <70; depressive symptoms that are due to effects of drugs of abuse or a neurological condition; a diagnosis of alcohol or other substance use disorders; current active suicidal ideation; repeated self-harm in the context of interpersonal conflict; having an immediate family member who works at the NIMH; and a serious medical condition such as epilepsy or heart disease.

Within this broader characterization study, participants are seen once per year for a full clinical assessment and are seen every four months between yearly follow up visits for questionnaires and neuroimaging scans. Recruitment for this broader study is ongoing. Information about the demographic characteristics of this sample can be found in Table 1 of the main paper.

**Interview Details and Additional Indices Created Using the Family History Interview**

The family history interview was created by modifying an existing family history interview for parent(s)/guardian(s) (Merikangas, 2006). To reduce time for administration, we deleted both physical and mental disorders that were not as relevant for our sample and only asked questions about treatment status and age of onset/offset of symptoms for depression and mania, rather than asking these follow up questions for every disorder. Additionally, rather than asking about each symptom of a disorder individually, we combined the common symptoms into a single probe that we used for each disorder.

For each participant, we generated a family tree of the a) biological, step, and adoptive parents, b) the fully biological, half-, step-, and adopted siblings, c) the biological aunts and uncles, d) the biological grandparents, e) the biological cousins, and f) biological nieces and nephews of the participant. Interviewees were then systematically asked if any of the listed family members had a formal diagnosis or some symptoms of the following disorders: Major Depressive Disorder, Mania/Bipolar Disorder, Anxiety Disorders, Psychosis, Obsessive Compulsive Disorder, Attention Deficit Hyperactivity Disorder, Eating Disorders, Post Traumatic Stress Disorder, Substance Use Disorders, Autism Spectrum Disorder, and Social Communication Disorder. For each disorder, we listed common symptoms to provide context for the informant. When discussing depression and mania symptoms, we asked whether the family member received treatment for their disorder and the age of onset/offset of symptoms. Finally, we asked if any family members had committed or attempted suicide.

Data obtained during the family history interview was consolidated into five indices. For each participant, we created variables answering the following five questions: 1) Does the participant have a family member who has attempted suicide (Yes/No)? 2) Does the participant have a family member who has completed suicide (Yes/No)? 3) How many family members with either a formal diagnosis or some symptoms of depression does the participant have? 4) Does the participant have a biological parent with either a formal diagnosis or some symptoms of depression (Yes/No)? 5) Does the participant have an immediate biological family member (parent or sibling) with either a formal diagnosis or some symptoms of depression (Yes/No)? This final variable was how we defined having a family history of depression in the rest of our analyses.

**Measures**

On the Mood and Feelings Questionnaire (MFQ) each of 13 items are self-rated using a 3-point scale. The psychometric properties of this scale have been previously evaluated (Thabrew, Stasiak, Bavin, Frampton, & Merry, 2018). We chose the MFQ because it a) has high internal reliability and criterion validity and b) captures central cognitive and affective depressive symptoms. The MFQ was presented as an online questionnaire in English and the score was quantified as the sum of all items; a higher score represents a more depressed mood.

The Child and Adolescent Survey of Experiences (CASE) is a 38-item list of possible events for which the respondent answers “yes” or “no.” Each affirmative response prompts a 6-point valence rating no neutral option) from “really good” to “really bad.” The respondent had the option to add up to 5 additional events of their own. We selected the CASE because of its high validity, capacity to detect associations between adolescent depression and negative events, and capture of both positive *and* negative events (Allen, Rapee, & Sandberg, 2012). We quantified adverse life events by summing the 3 negative CASE scores, (i.e. the life events that were rated from “a little bad,” to “really bad”).

**Deviations from the Preregistration**

There are a number of differences between our preregistration that was published in April 2020 and the methods and results presented in this paper. First and foremost, in our preregistration we stated that we would wait until October 2020 to run our analyses. Due to changes to our workload from the ongoing COVID-19 pandemic, we were not able to conduct the analyses in October, and instead held off on completing the analyses until spring of 2021. For this reason, our data includes participants who had visits between October 2020 and May 2021, which was different from what we originally planned. This allowed us to include more people with a one year follow up visit and a family history interview, and it also allowed us to further examine the impact of the COVID-19 pandemic on depressive episode duration and severity. To account for this, we included a variable in all of our analyses that represented whether or not the visit occurred after March of 2020 and would therefore overlap with the COVID-19 pandemic.

Next, in the second analysis section of our preregistration, we described how we would analyse the relationship between family history and future depressive severity, as measured by a score on the MFQ. We had planned to add in an additional model that examined the impact of an interaction between family history and stressful life events (CASE) on future MFQ scores. However, the CASE was only collected once yearly, and the MFQ scores were collected at many between visit intervals. Due to the overwhelming amount of missing CASE data given the differences in data collection procedures, we omitted this model from our analyses.

Next, in the third analysis section of our preregistration, we described our plan to use structural equation modelling to determine if family history influences the slope and intercept of the change in MFQ over time. However, due to the fact that each participant had a variable number of MFQ scores with varying amounts of time between each score, we were not able to complete structural equation modelling as planned. Instead, we fitted another linear mixed effect model that included a quadratic term for the interaction between family history and the amount of time between the two MFQ scores. This allowed us to examine the impact of family history on the slope and intercept of the change in MFQ over time as well as over quadratic time. These results can be found in the Supplemental Results section.

Finally, in our preregistration we stated that we would use repeated measures ANOVA to compare the RMSEs of the different models for both the weeks of depression and MFQ analyses. However, ANOVAs assume a Gaussian distribution of the response variable at each level, and the RMSEs were not Gaussian distributed. To make up for this, we instead conducted a fold-wise bootstrap and focused on a constrained set of informative comparisons for each analysis. Since there are only 125 unique bootstrap resamplings of five folds, we had to instead use eight-fold cross-validation, which gives 6,435 unique resamplings, so that we had enough resamplings to determine 99.9% confidence intervals, allowing us to correct for multiple comparisons. We additionally added analyses of MAE to supplement the analysis of RMSE and improve interpretability of the results.

**Weeks of Depression Analyses**

In our main analyses, we included some variables that were not present at baseline in order to control for them, but this does not reflect the information that would be available when making a prediction at the baseline visit. We also tested a set of models in which only information available at baseline was used, with the exception of a variable to control for the dates of the pandemic, as this remains a nuisance regressor in this analysis. Since the CASE was not collected at baseline, we did not use it in these models.

| **Model** | **Formula** |
| --- | --- |
| BSL_Null | Weeks of Depression ~ Antidepressants at Baseline + Other Meds at Baseline + Sex + Age + Post Pandemic |
| BSL_FH | Weeks of Depression ~ Family History + Antidepressants at Baseline + Other Meds at Baseline + Sex + Age + Post Pandemic |
| BSL_MFQ | Weeks of Depression ~ Baseline MFQ Score + Antidepressants at Baseline + Other Meds at Baseline + Sex + Age + Post Pandemic |
| BSL_MFQ+FH | Weeks of Depression ~ Baseline MFQ Score + Family History + Antidepressants at Baseline + Other Meds at Baseline + Sex + Age + Post Pandemic |

We examined the following pairwise differences with foldwise bootstrap confidence intervals. Differences in bold were presented in the main paper (Figure 1):

- **Null - FH**
- **Null - MFQ**
- **Null - MFQ+FH**
- Null - MFQ+FH+CASE
- **MFQ - MFQ+FH**
- MFQ - MFQ+FH+CASE
- MFQ+FH - MFQ+FH+CASE
- BSL_Null - BSL_FH
- BSL_Null - BSL_MFQ
- BSL_Null - BSL_MFQ+FH
- BSL_MFQ - BSL_MFQ+FH

This list contains 11 comparisons, so in order to protect against multiple comparisons we use 99.9% confidence intervals.

Due to errors with our online data collection interface, there were 13 individuals included in this analysis for whom we failed to collect one or two items for the Baseline MFQ. The scores for these individual items were imputed with multiple imputation.

**MFQ Analyses**

Calculating a simple pairwise correlation matrix for repeated measures produces correlations biased by within participant correlations. In order to avoid this problem we present separately correlations between terms with a single measure per participant (Baseline MFQ, Family History, Antidepressants at Baseline, and Other Meds at Baseline) and mean correlations across subjects for those terms that varied by visit pair (Next MFQ, Previous MFQ, Age, Interval, Pandemic, Inpatient) for the 114 subjects who contributed 3 or more visit pairs. We z-transformed the Pearson's correlations before averaging them and transformed them back for presentation. We also conducted one sample t-tests on the z-transformed values to determine significance. We applied a Bonferoni correction for the total number of comparisons between the two correlation matrices, which was 31.

As described in the differences from preregistration section, we included a model with a quadratic term for time between assessments (Interval Squared in model MFQ + FH_T2). In our weeks of depression analyses, we included baseline MFQ in order to control for the effects of baseline severity. Our analysis of the MFQ data is slightly different because we are fitting a slope across all pairs of MFQ scores. In our pre-registered models, we did not include a term for baseline severity, but as an exploratory analysis, we added models with baseline MFQ score in order to preserve a parallel structure with our analysis of weeks of depression.

| **Model** | **Formula** |
| --- | --- |
| MFQ + FH_T2 | Next MFQ ~ Family History*Interval + Family History:Interval Squared + Interval Squared + Previous MFQ + Antidepressants at Baseline + Other Meds at Baseline + Time Between the Two Scores + Inpatient + Previous Age + Sex + Pandemic + (1 + Interval \| Participant ID) |
| MFQ0 | Next MFQ ~ Baseline MFQ*Time Between the Two Scores + Antidepressants at Baseline + Other Meds at Baseline + Inpatient + Previous Age + Sex + Pandemic + (1 + Interval \| Participant ID) |
| MFQ0 + FH | Next MFQ ~ Baseline MFQ*Interval+ Family History:Interval + Family History + Antidepressants at Baseline + Other Meds at Baseline + Inpatient + Previous Age + Sex + Pandemic + (1 + Interval \| Participant ID) |
| MFQ + MFQ0 | Next MFQ ~ Baseline MFQ*Interval + Previous MFQ + Antidepressants at Baseline + Other Meds at Baseline + Inpatient + Previous Age + Sex + Pandemic + (1 + Interval \| Participant ID) |
| MFQ + MFQ0 + FH | Next MFQ ~ Baseline MFQ*Interval + Family History + Family History:Interval + Previous MFQ + Antidepressants at Baseline + Other Meds at Baseline + Time Between the Two Scores + Inpatient + Previous Age + Sex + Pandemic + (1 + Interval \| Participant ID) |

We examined the following pairwise differences with foldwise bootstrap confidence intervals. Differences in bold were presented in the main paper (Figure 2):

- **Null - FH**
- **Null - MFQ**
- **Null - MFQ+FH**
- Null - MFQ0
- Null - MFQ0+MFQ
- MFQ0 - MFQ0+FH
- MFQ0 - MFQ
- **MFQ - MFQ+FH**
- MFQ - MFQ+FH_T2
- MFQ+FH - MFQ+FH_T2
- MFQ - MFQ0+MFQ
- MFQ - MFQ0+MFQ+FH

This list contains 12 comparisons, so in order to protect against multiple comparisons we use 99.9% confidence intervals. We tested the benefit of including Interval Squared with a one-sided bootstrap t-test testing the null hypothesis that the change in RMSE is greater than 1.

Our main analysis calculates the RMSE for each fold weighted by the number of pairs of visits contributed by each participant. This could overrepresent results from participants with many pairs of visits, so as a sensitivity analysis, we repeated the analysis with RMSE for each fold unweighted by the number of visits each participant contributed.

Due to errors with our online data collection interface, there were 120 pairs of scores (out of a total of 1,311 pairs) from 39 participants in which one or two items were not collected on at least one of the MFQs in the pair. The scores for these individual items were imputed with multiple imputation.

**Sensitivity Analyses**

We conducted several analyses on the Weeks of Depression and MFQ datasets to determine if our results were robust to methodological choices. In the first sensitivity analysis, we used only a family history of a formal diagnosis of depression (FHD-dx) as opposed to a formal diagnosis and/or “some symptoms” to predict weeks of depression and MFQ score. In the second sensitivity analysis we used a family history of anxiety (FHA), both formal diagnosis and “some symptoms,” to predict weeks of depression and MFQ score. In the third sensitivity analysis we used the original FHD variable but excluded all current and former inpatients to remove any possible impact of treatment resistant depression in the analyses. Identical analyses to the original analyses were run using the updated variables and or datasets.

In our fourth sensitivity analysis, we also used our 8-fold cross-validation and foldwise bootstrap to fit elastic net regressions and extremely randomized trees regressions to the weeks of depression and MFQ analyses. For the MFQ analyses, we took only the first pair of visits for each individual to avoid the complications of data with multiple results per participant. For comparison, we also used a linear regression with 8-fold cross-validation and foldwise bootstrap for this single pair MFQ data. Since this was an exploratory analysis, we did not do any nested cross-validation for hyperparameter optimization, instead using the defaults for these methods from Scikit-learn (version 0.24.2, Pedregosa et al., 2011).

In our fifth sensitivity analysis, we predicted parent-report MFQ instead of self-report MFQ with FHD.

In our sixth sensitivity analysis, we wanted to confirm that FHD was not predictive of MFQ at a subset of the intervisit intervals in our sample. We subset the data at terciles of the intervisit interval giving us short intervals (1-15 days), medium intervals (16-77 days), and long intervals (77-518 days), additionally we used just the first and last visit to give the longest possible intervisit intervals (Table S21 for characteristics of each subsample). For the short, medium, and long interval subsets, we conducted the analysis with mixed effects models since these subsets still had multiple visits per participant. For the first and last visit analysis, we used linear models.

We conducted an exploratory analysis in order to confirm that FHD does not have an impact on trajectories of depressive severity. We fit the following mixed effects model to the MFQ data:

Current MFQ ~ FHD*Time since baseline + Antidepressants at Baseline + Other Meds at Baseline + Inpatient + Previous Age + Sex + Pandemic + (1 + Time since baseline| Participant ID) + (1 | Visit number).

This model is different from those in the main analysis because we are trying to extract the influence of family history of depression on the overall trend of MFQ scores, not predict subsequent MFQ scores. If FHD influences trajectories, we would expect to see a significant FHD - Time since baseline interaction. This model was also used to generate predicted MFQ scores for Figure S1.

In order to test if the distribution of subject level trajectories might differ by FHD in a way not detected by the mixed effects model, we fit a model without FHD:

Current MFQ ~ Time since baseline + Antidepressants at Baseline + Other Meds at Baseline + Inpatient + Previous Age + Sex + Pandemic + (1 + Time since baseline| Participant ID) + (1 | Visit number).

We extracted the subject level estimates of the coefficient of Time since baseline, which should provide the best estimate of that trajectory while controlling for potential confounds. Additionally, we categorized the trajectories as worsening (Time since baseline beta > 0.5 MFQ points per year), improving (Time since baseline beta < -0.5 MFQ points per year), or flat (-0.5 < Time since baseline beta < 0.5) and performed a chi-squared test on the frequency of the three trajectory categories between those with and without an FHD.

To explore the relationship between FHD and individual depressive symptoms we ran a linear discriminant analysis (LDA) on individual items from the MFQ. We conducted the LDA on MFQ scores from baseline for the 129 individuals included in the MFQ analysis. We partitioned the dataset into a training (n = 79) and a testing dataset (n = 50) and ran the “lda” function from the R MASS package on the training dataset (v7.3-54, Venables & Ripley, 2002). We then predicted the FHD status of the participants in the test dataset and calculated the Matthews Correlation Coefficient (MCC; Chicco, Tötsch, & Jurman, 2021) of the trained linear model. MCC is essentially a measure of the correlation between the true labels and predicted labels, with values of 0 for chance performance and 1 for perfect performance. It is much better for assessing the performance of classification in the case of unbalanced classes than accuracy. We compared the MCC of the model on the test dataset to chance as determined by 10,000 simulations of randomly assigned labels.

**Power Analysis for Weeks of Depression**

Since our primary statistical test is a non-parametric 8-fold cross validation with exhaustive fold-wise bootstrap, we are unable to rely on a parametric power calculation as implemented in tools such as G*Power. Instead, we must determine the power of the analyses via simulation. Since our analysis is a comparison of distributions of residuals, our simulations need to create pairs of residual distributions. One distribution in each pair is the baseline or “null” distribution. To determine the power of our analysis, we need the other distribution in each pair to differ in some way, where the magnitude of the difference is the effect size. We call this distribution the “changed” distribution. For each simulation, we first create distributions for a large population, then calculate the difference in RMSE and MAE between the “null” and “changed” residual distributions in the large population. This gives us our ground truth for that simulation, and then we draw samples of various sizes from this large population. Repeating this process many times, we can determine how likely it is that our approach (8-fold cross validation with fold-wise bootstrap) will find a significant difference between RMSE and MAE across a range of effect sizes. This relationship is our power analysis.

For the weeks of depression analysis, we created 2,000 simulations of 10,000 participants each. The distribution of “null” residuals was drawn from a kernel density estimate fitted to the distribution of residuals from the MFQ + Family History model. For each population, we also created an effect distribution with a mean and standard deviation. The mean and standard deviation for the effect distribution were drawn from uniform distributions; 0.25-3 for the mean and 0.5-1.0 for the standard deviation. The “changed” distribution was created by multiplying each element in the null distribution by the corresponding element from the effect distribution. We then transformed the null and changed residuals into absolute error and squared error in order to calculate population level effect sizes (Cohen’s d) for the difference between the two residuals. We found that calculating Cohen’s d on the squared error corresponded better with MAE effect sizes than using the root square error. Samples of 36, 72, 200, and 1,000 participants were drawn from each simulated population. We used samples of 72 since this was the size of our sample, samples of 36 were used because this was half of our sample size, and 200 and 1,000 participant samples were drawn to confirm that the power-curves shifted appropriately with increased sample size. The fold-wise bootstrap was carried out within each sample to determine the confidence intervals around the difference in MAE and RMSE. We then created power-curves by binning the absolute value of the population effect size and calculating the percent of significant differences found within each bin for each metric and sample size. For comparison, we also calculated effect size and power for an F-test of change in *r^2^* using G*Power (Faul, Erdfelder, Lang, & Buchner, 2007).

**Power Analysis for MFQ**

The procedure for the MFQ analysis was similar, except that simulations were conducted to preserve the structure of multiple observations being collected for each participant. These structured residual distributions were created by fitting a mixed effects model (residual ~ 1 + (1|subject)) to the residuals of the MFQ + Family History model and then simulating the appropriate population size with SIMR (Green & MacLeod, 2016). The changed distribution corresponding to each null distribution was simulated by adjusting the residual variance and generating a new set of simulated residuals with SIMR. The effects were drawn from a uniform distribution: 0.25 - 3.35. We again created 2,000 simulations of 10,000 participants each. Samples of 65, 129, 300, and 1,000 were drawn from each population. Note that the use of SIMR preserves the distribution of the number of assessments per individual that we observed in our sample (Figure S6). We used samples of 129 since this was the size of our sample, samples of 65 were used because this was half of our sample size, and 300 and 1,000 participant samples were drawn to confirm that the power-curves shifted appropriately with increased sample size. Fold-wise bootstrap and creation of power-curves was carried out as described above.

**Supplemental Results**

**Weeks of Depression Analyses**

Examining the pairwise correlations between terms (Figure S2), we see that weeks of depression is correlated with baseline severity and stressful life events as assessed by the CASE. There are also significant correlations between medication use at baseline and follow up.

Since the baseline models use fewer variables, there are more participants with sufficient data to include, 92 in total (demographic information is in Table S15). In both the baseline MFQ model and baseline MFQ + family history model with this bigger dataset, other medications at baseline were no longer significantly associated with weeks of depression, but the rest of the correlations were the same between the two datasets (Table S2, Table S3, Table S18, and Table S19). However, in the family history only model with this larger dataset, family history was not significantly associated with weeks of depression, which was a different result than with our original dataset (Table S5 and Table S17). The differences in predictive performance between models that use only those variables available at baseline tend to have slightly smaller bootstrap confidence intervals (Figure S5), likely due to the inclusion of 20 additional participants. However, all of the results from the models in the main paper are essentially the same. Inclusion of family history does not reduce the MAE of any models by more than 1 week (one-sided bootstrap t-test, p<1.6x10^-4) and none of the models have an RMSE less than 10 weeks (one-sided bootstrap t-test, p<1.6x10^-4).

**MFQ Analyses**

We examined the pairwise correlations between terms from the MFQ Analyses in two sets. The first includes those terms for which there is a single value per participant (Figure S3), from which we see the only significant correlation is between baseline medications. For the terms that vary per assessment pair (Figure S4), we see that there is on average a modest correlation between subsequent MFQs (Mean ± SE correlation = 0.22 ± 0.06, t_113_=3.92, p = 0.00015). Strong correlations are present between age and onset of the pandemic due to the occurrence of the pandemic in the later half of our data collection period. The inpatient unit at the NIH was closed during the COVID-19 pandemic, giving rise to the negative correlations between inpatient and age and inpatient and pandemic.

We have compared two sets of models to determine if adding a quadratic term for time improved prediction, MFQ - MFQ+FH_T2 and MFQ+FH - MFQ+FH_T2 (Figure S7B). In neither case is the RMSE reduced by more than 1 (one-sided bootstrap t-test, p<1.6x10^-4).

The 99.9% bootstrap confidence interval around the difference between the null and MFQ models indicates that the MFQ model improves on the null by at least 1 point, but less than 3 points. The minimum clinically relevant difference on the short MFQ, as used in this study, is 6 points, and, thus, the MFQ improves the performance of the null model by less than half of that.

In our pre-registered models, we did not include a term for baseline severity, but as an exploratory analysis, we added models with baseline MFQ score in order to preserve a parallel structure with our analysis of weeks of depression. As is clear from the confidence intervals (Figure S7B), including the baseline MFQ score did reduce the RMSE, but none of the models that included it improved on the RMSE of the Null model by even 3 points, half of the clinically meaningful difference on the MFQ.

Analyses with an unweighted RMSE in which each participant only contributes a single value to the fold-wise RMSE did not differ from the results with the weighted RMSE in any meaningful way, though the bootstrap confidence intervals are slightly larger (Figure S17).

**Sensitivity Analyses**

In order to verify that our findings were robust to our methodological choices we ran several sensitivity analyses.

The first sensitivity analyses replaced the original FHD variable with a variable that indicated only family history of formal diagnosis of depression (FHD-dx), excluding those with only “some symptoms.” Because this variable was quite similar to the original FHD variable, we expected similar results to the original analyses. As anticipated, both the Weeks of Depression (Figure S8) and MFQ analyses (Figure S9) conducted with this new FHD-dx variable did not show any significant results.

The second sensitivity analyses replaced the original FHD variable with a variable that indicated family history of anxiety (FHA). Notably, this variable included both those with a formal diagnosis as well as those with “some symptoms.” This variable was constructed identically to the original FHD variable. Because the connection between family history of anxiety and depression is not as well established as FHD and depression, we expected similar results to the original analyses, if not less significant. As anticipated, both the Weeks of Depression (Figure S10) and MFQ analyses (Figure S11) conducted with this new FHA variable did not show any significant results.

The third sensitivity analysis excluded all participants who were current or former inpatients to avoid the impact of treatment resistance on the analyses. Because our cohort does not select for treatment resistance in recruitment for characterization or inpatient enrolment, we expected that excluding inpatients would not have a large impact on the results. As anticipated, both the Weeks of Depression (Figure S12) and MFQ analyses (Figure S13) conducted without current or former inpatients did not show any significant results.

In the fourth sensitivity analysis we used elastic net regression and randomized trees regression instead of unregularized linear models. In the two primary model comparisons with the addition of family history (Null - FH and MFQ - MFQ + FH), all three methods performed similarly (Figure S14).

In the fifth sensitivity analysis we used parent-report MFQ instead of self-report MFQ to verify that our result is robust to reporter. We collected fewer parent-report MFQ scores, so this analysis had fewer subjects (122 instead of 129), and fewer assessments (925 instead of 1,439). Demographics of the samples for our main depression severity analysis were overall quite similar though (Table S20). As in our main analysis, FHD did not improve prediction of depression severity based on parent-report MFQ scores (Figure S15).

In the sixth sensitivity analysis, we examined different time intervals: short (1-15 days), medium (16-77 days), and long (77-518 days), as well as the first and last visits. FHD did not improve prediction of depression severity in subsets of visits in any of these time scales (Figure S16). However, the Null - MFQ comparison does confirm our expectation that previous MFQ improves prediction more when there is a shorter interval between visits.

If FHD influences trajectories, we would expect to see a significant FHD - Time since baseline interaction, but instead we found that neither FHD, nor the interaction of FHD with Time since baseline were significant (FHD: beta = 1.16, 95%CI = [-1.37, 3.70], T_118.42_ = 118.42, p = 0.37; FHD:Time since baseline: beta = -0.261, 95%CI = [-1.72, 1.20], T_92.44_ = -0.351, p = 0.726).

In order to test if the distribution of subject level trajectories might differ by FHD in a way not detected by the mixed effects model, we fit a model without FHD:

Current MFQ ~ Time since baseline + Antidepressants at Baseline + Other Meds at Baseline + Inpatient + Previous Age + Sex + Pandemic + (1 + Time since baseline| Participant ID) + (1 | Visit number)

We extracted the subject level estimates of the coefficient of Time since baseline, which should provide the best estimate of that trajectory while controlling for potential confounds. We performed a t-test comparing these coefficients between individuals with and without an FHD and did not find a significant difference (T_127_ = 0.48, p-value = 0.63). Additionally, we categorized the trajectories as worsening (Time since baseline beta > 0.5 MFQ points per year), improving (Time since baseline beta < -0.5 MFQ points per year), or flat (-0.5 < Time since baseline beta < 0.5) and performed a chi-squared test on the frequency of the three trajectory categories between those with and without an FHD. We did not find a significant difference in the distribution of trajectories when categorized in this way (Χ^2^ = 0.038, p-value = 0.98).

Finally, we conducted an LDA to see if patterns of depressive symptoms at baseline differed based on the presence of FHD. We did not find that the LDA model performed better than chance in a test dataset (MCC = 0.12, one-sided p-value = 0.18). This indicates that there is not a systematic difference in MFQ item responses between the FHD and no FHD groups. With a larger sample size or different measures of depression symptoms, there may be a difference in the pattern of symptoms as a factor of FHD, but we did not find evidence for this in our sample.

**Power Analysis for Weeks of Depression**

Our simulations show that we were underpowered to detect a difference in model performance of 2 weeks. We had an 80.0% chance to detect a significant effect with an absolute population Cohen’s d between 0.43 and 0.46 and a sample of 72 participants when using MAE (Figure S19). This corresponds to an absolute mean MAE difference of 7.01 weeks assuming a pooled standard deviation of 15.77 weeks. Similarly, we found an 87.0% chance to detect a significant effect with an absolute population Cohen’s d between 0.43 and 0.46 and a sample of 72 participants when using RMSE. This corresponds to an average RMSE difference of 9.6 weeks. A minimally clinically significant difference of 2 weeks MAE corresponds to an absolute Cohen’s d of 0.056 in our simulations, which we had only a 5.3% power to detect. A difference of 2 weeks RMSE corresponds to an absolute Cohen’s d of 0.11 in our simulations, which we had only a 2.3% power to detect. This result is in concordance with the width of the bootstrap confidence intervals in our primary analysis of the Weeks of depression (Figure 1).

If we had simply performed a comparison of linear models to determine if including family history of depression improved the model fit via an F-test we would have had 80% power to detect a 11.25% improvement in r^2^, a difference in model fit corresponding to 3.68 weeks (calculations below). Our specified minimally clinically significant difference of 2 weeks corresponds to 3.32% of the variance explained by the MFQ+FHD model. If we use 3.32% as the input f^2^ to a power calculation in G*Power, we find that it would require a sample of 246 participants to have 80% power to detect a 3.32% difference in r^2^. Here is how we related weeks to percent variance explained: The MFQ+FHD model fitted to the full dataset had an r^2^ of 0.365 and the total variance of weeks of depression was 329.83 weeks^2^. Treating r^2^ as percent variance explained, this means that the MFQ+FHD model explained 120.38 weeks^2^ of variance (329.83 * 0.365 = 120.38). 11.25% of the 120.38 weeks^2^ of variance explained by the MFQ+FHD model corresponds to 13.54 weeks^2^ (120.38 * 0.1125 = 13.54) or 3.68 weeks. From this calculation we see that a comparison of linear models would be able to detect a difference in model fit of 3.68 weeks. If we instead work backwards from our specified minimally clinically significant difference of 2 weeks, this corresponds to 4 weeks^2^ of variance, or 3.32% of the variance explained by the MFQ+FH model (4 / 120.38 = 0.0332). If we then use 3.32% as the input f^2^ to a power calculation in G*Power, we find that It would require a sample of 246 participants to have 80% power to detect a 3.32% difference in r^2^.

**Power Analysis for MFQ**

For the MFQ analysis, our simulations indicate that we were sufficiently powered to detect effects well below the minimally significant clinical difference for the MFQ of 6 points (Figure S18). We had an 81.3% chance to detect a significant effect with an absolute population Cohen's d between 0.19 and 0.22 and a sample of 129 participants when using MAE. This corresponds to an absolute mean MAE difference of 0.58 assuming a pooled standard deviation of 2.81, which we based on our simulations. Similarly, we found a 79.3% chance to detect an absolute population Cohen's d between 0.19 and 0.22 and a sample of 129 participants when using RMSE. This corresponds to an average RMSE difference of 0.72.

Given the structure of the MFQ analysis with pairs of assessments nested within individuals, a mixed effects model would be the most appropriate typical analytical approach. It is difficult to assess the power of mixed effects models in the same way as linear models, so we instead conducted a power analysis on a linear model fit to a single pair of visits per individual as an assessment of the lower bound of the power of the mixed effects model which can take all of the assessment pairs into account. We found that even this lower bound on the power of a mixed effects model was sufficiently powered to detect the difference in model fit of 0.91 MFQ points, well below the minimally significant clinical difference for the MFQ of 6 points. We constructed the dataset for this analysis by selecting a random pair of visits from each individual. We then fit a linear model with the fixed effects terms of the MFQ+FHD model: Next MFQ ~ Previous MFQ + FHD*Interval + Antidepressants at Baseline + Other Meds at Baseline + Inpatient + Previous Age + Sex + Pandemic. This model had an r^2^ of 0.58 when fit to the data and the total variance of “Next MFQ” scores was 44.88 (MFQ points)^2^, thus the MFQ+FHD model explained 25.86 (MFQ points)^2^ of variance. An F-test of the significance of one predictor out of 11 total predictors in 129 participants has 80% power to detect a 3.22% increase in r^2^. 3.22% of 25.86 (MFQ points)^2^ is 0.83 (MFQ points)^2^ or 0.91 MFQ points.

**Supplemental References**

Allen, J. L., Rapee, R. M., & Sandberg, S. (2012). Assessment of Maternally Reported Life Events in Children and Adolescents: A Comparison of Interview and Checklist Methods. *Journal of Psychopathology and Behavioral Assessment*, *34*(2), 204–215.

Chicco, D., Tötsch, N., & Jurman, G. (2021). The Matthews correlation coefficient (MCC) is more reliable than balanced accuracy, bookmaker informedness, and markedness in two-class confusion matrix evaluation. *BioData Mining*, *14*, 13.

Faul, F., Erdfelder, E., Lang, A.-G., & Buchner, A. (2007). G*Power 3: A flexible statistical power analysis program for the social, behavioral, and biomedical sciences. *Behavior Research Methods*, *39*(2), 175–191.

Green, P., & MacLeod, C. J. (2016). SIMR: An R package for power analysis of generalized linear mixed models by simulation. *Methods in Ecology and Evolution*, *7*(4), 493–498.

Merikangas, K. R. (2006, August 30). Family History Interview. Genetic Epidemiology Research Branch (GEB-SDGE).

Pedregosa, F., Varoquaux, G., Gramfort, A., Michel, V., Thirion, B., Grisel, O., Blondel, M., et al. (2011). Scikit-learn: Machine Learning in Python. *MACHINE LEARNING IN PYTHON*, 6.

Thabrew, H., Stasiak, K., Bavin, L., Frampton, C., & Merry, S. (2018). Validation of the Mood and Feelings Questionnaire (MFQ) and Short Mood and Feelings Questionnaire (SMFQ) in New Zealand help‐seeking adolescents. *International Journal of Methods in Psychiatric Research*, *27*(3). Retrieved July 9, 2021, from https://onlinelibrary.wiley.com/doi/10.1002/mpr.1610

Venables, & Ripley. (2002). MASS v7.3-54. Retrieved October 14, 2021, from https://cran.r-project.org/web/packages/MASS/citation.html

**Supplemental Tables**

| **Weeks of Depression Analyses** | | | | |
| --- | --- | --- | --- | --- |
| **Table S1: Linear Regression Results for Null Model**  Weeks of Depression ~ Antidepressants at Baseline + Other Meds at Baseline + Antidepressants at FU + Other Meds at FU + Inpatient Status + Sex + Age at Baseline + Post Pandemic | | | | |
| **Variable** | **Estimate** | **Std. Error** | **t-Value** | **p-Value** |
| Antidepressants at Baseline | 1.080 | 5.384 | 0.200 | 0.842 |
| Other Meds at Baseline | -10.309 | 5.930 | -1.738 | 0.087 |
| Antidepressants at FU | 7.761 | 5.376 | 1.444 | 0.154 |
| Other Meds at FU | 2.355 | 6.253 | 0.377 | 0.708 |
| Inpatient Status | 6.640 | 5.412 | 1.227 | 0.224 |
| Sex (Male) | -3.593 | 4.997 | -0.719 | 0.475 |
| Age | 1.193 | 1.657 | 0.720 | 0.474 |
| Post Pandemic | 5.172 | 4.628 | 1.118 | 0.268 |
| **Model Statistics** | | | | |
| **Residual Standard Error** | **Multiple R Squared** | **Adjusted R Squared** | **F Statistic** | **Model p-Value** |
| 18.06 on 63 df | 0.135 | 0.025 | 1.225 on 8 and 63 df | 0.300 |

| **Weeks of Depression Analyses** | | | | |
| --- | --- | --- | --- | --- |
| **Table S2: Linear Regression Results for MFQ Model**  Weeks of Depression ~ Baseline MFQ Score + Antidepressants at Baseline + Other Meds at Baseline + Antidepressants at FU + Other Meds at FU + Inpatient Status + Sex + Age at Baseline + Post Pandemic | | | | |
| **Variable** | **Estimate** | **Std. Error** | **t-Value** | **p-Value** |
| Baseline MFQ Score | 1.164 | 0.317 | 3.669 | <0.001*** |
| Antidepressants at Baseline | 6.609 | 5.146 | 1.284 | 0.204 |
| Other Meds at Baseline | -13.271 | 5.478 | -2.423 | 0.018* |
| Antidepressants at FU | 4.057 | 5.015 | 0.809 | 0.422 |
| Other Meds at FU | 2.766 | 5.715 | 0.484 | 0.630 |
| Inpatient Status | 2.251 | 5.087 | 0.442 | 0.660 |
| Sex (Male) | -4.884 | 4.579 | -1.067 | 0.290 |
| Age | 0.130 | 1.541 | 0.085 | 0.933 |
| Post Pandemic | 5.648 | 4.230 | 1.335 | 0.187 |
| **Model Statistics** | | | | |
| **Residual Standard Error** | **Multiple R Squared** | **Adjusted R Squared** | **F Statistic** | **Model p-Value** |
| 16.5 on 62 df | 0.289 | 0.186 | 2.799 on 9 and 62 df | 0.008** |

| **Weeks of Depression Analyses** | | | | |
| --- | --- | --- | --- | --- |
| **Table S3: Linear Regression Results for MFQ + FH Model**  Weeks of Depression ~ Baseline MFQ Score + Family History + Antidepressants at Baseline + Other Meds at Baseline + Antidepressants at FU + Other Meds at FU + Inpatient Status + Sex + Age at Baseline + Post Pandemic | | | | |
| **Variable** | **Estimate** | **Std. Error** | **t-Value** | **p-Value** |
| Baseline MFQ Score | 1.180 | 0.302 | 3.904 | <0.001*** |
| Family History | 11.384 | 4.208 | 2.705 | 0.009** |
| Antidepressants at Baseline | 6.073 | 4.906 | 1.238 | 0.220 |
| Other Meds at Baseline | -12.472 | 5.227 | -2.386 | 0.020* |
| Antidepressants at FU | 3.765 | 4.779 | 0.788 | 0.434 |
| Other Meds at FU | 2.608 | 5.445 | 0.479 | 0.634 |
| Inpatient Status | 1.890 | 4.848 | 0.390 | 0.698 |
| Sex (Male) | -4.849 | 4.363 | -1.112 | 0.271 |
| Age | -0.749 | 1.504 | -0.498 | 0.620 |
| Post Pandemic | 5.929 | 4.031 | 1.471 | 0.147 |
| **Model Statistics** | | | | |
| **Residual Standard Error** | **Multiple R Squared** | **Adjusted R Squared** | **F Statistic** | **Model p-Value** |
| 15.72 on 61 df | 0.365 | 0.261 | 3.508 on 10 and 61 df | 0.001** |

| **Weeks of Depression Analyses** | | | | |
| --- | --- | --- | --- | --- |
| **Table S4: Linear Regression Results for MFQ + FH + CASE Model**  Weeks of Depression ~ Baseline MFQ Score + Family History*Stressful Life Events + Antidepressants at Baseline + Other Meds at Baseline + Antidepressants at FU + Other Meds at FU + Inpatient Status + Sex + Age at Baseline + Post Pandemic | | | | |
| **Variable** | **Estimate** | **Std. Error** | **t-Value** | **p-Value** |
| Baseline MFQ Score | 0.923 | 0.322 | 2.868 | 0.006** |
| Family History | 7.581 | 7.269 | 1.043 | 0.301 |
| Stressful Life Events | 0.414 | 1.256 | 0.330 | 0.743 |
| Antidepressants at Baseline | 5.599 | 5.130 | 1.091 | 0.280 |
| Other Meds at Baseline | -10.740 | 5.158 | -2.082 | 0.042* |
| Antidepressants at FU | 4.004 | 4.938 | 0.811 | 0.421 |
| Other Meds at FU | 0.949 | 5.350 | 0.177 | 0.860 |
| Inpatient Status | 3.843 | 4.920 | 0.781 | 0.438 |
| Sex (Male) | -3.970 | 4.287 | -0.926 | 0.358 |
| Age | -0.944 | 1.467 | -0.644 | 0.522 |
| Family History*Stressful Life Events | 0.901 | 1.333 | 0.676 | 0.502 |
| Post Pandemic | 5.565 | 3.928 | 1.417 | 0.162 |
| **Model Statistics** | | | | |
| **Residual Standard Error** | **Multiple R Squared** | **Adjusted R Squared** | **F Statistic** | **Model p-Value** |
| 15.3 on 59 df | 0.418 | 0.300 | 3.538 on 12 and 59 df | <0.001*** |

| **Weeks of Depression Analyses** | | | | |
| --- | --- | --- | --- | --- |
| **Table S5: Linear Regression Results for FH Model**  Weeks of Depression ~ Family History + Antidepressants at Baseline + Other Meds at Baseline + Antidepressants at FU + Other Meds at FU + Inpatient Status + Sex + Age at Baseline + Post Pandemic | | | | |
| **Variable** | **Estimate** | **Std. Error** | **t-Value** | **p-Value** |
| Family History | 11.059 | 4.666 | 2.370 | 0.021* |
| Antidepressants at Baseline | 0.484 | 5.203 | 0.093 | 0.926 |
| Other Meds at Baseline | -9.493 | 5.734 | -1.656 | 0.103 |
| Antidepressants at FU | 7.527 | 5.191 | 1.450 | 0.152 |
| Other Meds at FU | 2.196 | 6.037 | 0.364 | 0.717 |
| Inpatient Status | 6.350 | 5.225 | 1.215 | 0.229 |
| Sex (Male) | -3.542 | 4.823 | -0.734 | 0.466 |
| Age | 0.353 | 1.638 | 0.215 | 0.830 |
| Post Pandemic | 5.439 | 4.468 | 1.217 | 0.228 |
| **Model Statistics** | | | | |
| **Residual Standard Error** | **Multiple R Squared** | **Adjusted R Squared** | **F Statistic** | **Model p-Value** |
| 17.43 on 62 df | 0.206 | 0.091 | 1.792 on 9 and 62 df | 0.088 |

| **MFQ Analyses** | | | | | |
| --- | --- | --- | --- | --- | --- |
| **Table S6: Linear Mixed Effect Results for Model 2 (Null Model + Previous MFQ + Family History)**    MFQ Score ~ Family History*Time Between + Previous MFQ + Antidepressants at Baseline + Other Meds at Baseline + Inpatient + Sex + Age + Post Pandemic, random = Time Between \| Participant ID | | | | | |
| **Variable** | **Value** | **Std. Error** | **DF** | **t-Value** | **p-Value** |
| Family History | 0.702 | 0.549 | 124 | 1.278 | 0.204 |
| Time Between the two scores | -0.001 | 0.004 | 1175 | -0.232 | 0.817 |
| Previous MFQ Score | 0.581 | 0.022 | 1175 | 25.964 | <0.001*** |
| Inpatient Status | -0.765 | 0.451 | 1175 | -1.697 | 0.090 |
| Age | -0.173 | 0.111 | 1175 | -1.561 | 0.119 |
| Sex (Male) | -1.334 | 0.444 | 124 | -3.007 | 0.003** |
| Antidepressants at Baseline | 0.005 | 0.445 | 124 | 0.010 | 0.992 |
| Other Medications at Baseline | 0.389 | 0.495 | 124 | 0.786 | 0.433 |
| Post Pandemic | -0.353 | 0.320 | 1175 | -1.102 | 0.271 |
| Interaction between family history and time between | -0.002 | 0.004 | 1175 | -0.434 | 0.664 |
| **Model Statistics** | | | | | |
| **AIC** | | **BIC** | | **logLik** | |
| 7596.782 | | 7674.322 | | -3783.391 | |

| **MFQ Analyses** | | | | | |
| --- | --- | --- | --- | --- | --- |
| **Table S7: Linear Mixed Effect Results for MFQ0 + MFQ + FH Model**  MFQ Score ~ Time Between* Baseline MFQ + Family History + Family History:Time Between + Previous MFQ + Antidepressants at Baseline + Other Meds at Baseline + Inpatient + Sex + Age + Post Pandemic, random = Time Between \| Participant ID | | | | | |
| **Variable** | **Estimate** | **Std. Error** | **DF** | **t-Value** | **p-Value** |
| Antidepressants at Baseline | 0.452 | 0.440 | 123 | 1.027 | 0.306 |
| Time Between the Two Scores | 0.002 | 0.005 | 1174 | 0.418 | 0.676 |
| Baseline MFQ Score | 0.191 | 0.037 | 123 | 5.192 | <0.001*** |
| Family History | 0.386 | 0.523 | 123 | 0.737 | 0.463 |
| Previous MFQ | 0.537 | 0.024 | 1174 | 22.637 | <0.001*** |
| Inpatient | -0.835 | 0.441 | 1174 | -1.892 | 0.059 |
| Age | -0.163 | 0.108 | 1174 | -1.510 | 0.131 |
| Sex (Male) | -1.560 | 0.432 | 123 | -3.609 | <0.001*** |
| Other Meds at Baseline | -0.068 | 0.487 | 123 | -0.140 | 0.889 |
| Post Pandemic | -0.443 | 0.316 | 1174 | -1.403 | 0.161 |
| Interaction between Time Between Scores and Baseline MFQ Score | 0.000 | 0.000 | 1174 | -0.948 | 0.344 |
| Interaction between Time Between and Family History | -0.001 | 0.004 | 1174 | -0.257 | 0.797 |
| **Model Statistics** | | | | | |
| **AIC** | | **BIC** | | **logLik** | |
| 7589.235 | | 7677.088 | | -3777.618 | |

| **MFQ Analyses** | | | | | |
| --- | --- | --- | --- | --- | --- |
| **Table S8: Linear Mixed Effect Results for Null Model**    MFQ Score ~ Antidepressants at Baseline + Other Meds at Baseline + Inpatient + Sex + Age + Post Pandemic + Time Between, random = Time Between \| Participant ID | | | | | |
| **Variable** | **Value** | **Std. Error** | **DF** | **t-Value** | **p-Value** |
| Antidepressants at Baseline | -0.061 | 1.038 | 125 | -0.059 | 0.953 |
| Time Between the two scores | -0.005 | 0.002 | 1177 | -1.926 | 0.054 |
| Inpatient Status | -0.315 | 0.528 | 1177 | -0.597 | 0.551 |
| Age | -0.554 | 0.207 | 1177 | -2.671 | 0.008** |
| Sex (Male) | -3.380 | 1.034 | 125 | -3.269 | 0.001** |
| Other Medications at Baseline | 0.613 | 1.161 | 125 | 0.529 | 0.598 |
| Post Pandemic | -1.068 | 0.432 | 1177 | -2.473 | 0.014* |
| **Model Statistics** | | | | | |
| **AIC** | | **BIC** | | **logLik** | |
| 7945.064 | | 8007.124 | | -3960.532 | |

| **MFQ Analyses** | | | | | |
| --- | --- | --- | --- | --- | --- |
| **Table S9: Linear Mixed Effect Results for Model 1 (Null Model + Previous MFQ)**    MFQ Score ~ Previous MFQ + Antidepressants at Baseline + Other Meds at Baseline + Inpatient + Sex + Age + Post Pandemic + Time Between, random = Time Between \| Participant ID | | | | | |
| **Variable** | **Value** | **Std. Error** | **DF** | **t-Value** | **p-Value** |
| Antidepressants at Baseline | 0.031 | 0.443 | 125 | 0.070 | 0.945 |
| Time Between the two scores | -0.002 | 0.002 | 1176 | -1.209 | 0.227 |
| Previous MFQ Score | 0.584 | 0.022 | 1176 | 26.200 | <0.001*** |
| Inpatient Status | -0.762 | 0.450 | 1176 | -1.692 | 0.091 |
| Age | -0.158 | 0.110 | 1176 | -1.441 | 0.150 |
| Sex (Male) | -1.351 | 0.442 | 125 | -3.058 | 0.003** |
| Other Medications at Baseline | 0.404 | 0.493 | 125 | 0.819 | 0.414 |
| Post Pandemic | -0.368 | 0.319 | 1176 | -1.152 | 0.250 |
| **Model Statistics** | | | | | |
| **AIC** | | **BIC** | | **logLik** | |
| 7585.76 | | 7652.982 | | -3779.88 | |

| **MFQ Analyses** | | | | | |
| --- | --- | --- | --- | --- | --- |
| **Table S10: Linear Mixed Effect Results for Model 3 (Null Model + Family History)**    MFQ Score ~ Family History*Time Between + Antidepressants at Baseline + Other Meds at Baseline + Inpatient + Sex + Age + Post Pandemic, random = Time Between \| Participant ID | | | | | |
| **Variable** | **Value** | **Std. Error** | **DF** | **t-Value** | **p-Value** |
| Family History | 1.297 | 1.128 | 124 | 1.150 | 0.252 |
| Time Between the two scores | -0.004 | 0.005 | 1176 | -0.814 | 0.416 |
| Inpatient Status | -0.318 | 0.529 | 1176 | -0.602 | 0.547 |
| Age | -0.565 | 0.207 | 1176 | -2.725 | 0.007** |
| Sex (Male) | -3.293 | 1.034 | 124 | -3.185 | 0.002** |
| Antidepressants at Baseline | -0.117 | 1.036 | 124 | -0.113 | 0.911 |
| Other Medications at Baseline | 0.605 | 1.158 | 124 | 0.523 | 0.602 |
| Post Pandemic | -1.052 | 0.432 | 1176 | -2.433 | 0.015* |
| Interaction between family history and time between | -0.001 | 0.006 | 1176 | -0.193 | 0.847 |
| **Model Statistics** | | | | | |
| **AIC** | | **BIC** | | **logLik** | |
| 7954.322 | | 8026.704 | | -3963.161 | |

| **MFQ Analyses** | | | | | |
| --- | --- | --- | --- | --- | --- |
| **Table S11: Linear Mixed Effect Results for Model 4 (Quadratic Family History Model)**    MFQ Score ~ Family History* Time Between + Family History*Time^2 + Previous MFQ + Antidepressants at Baseline + Other Meds at Baseline + Inpatient + Sex + Age + Post Pandemic, random = Time Between \| Participant ID | | | | | |
| **Variable** | **Value** | **Std. Error** | **DF** | **t-Value** | **p-Value** |
| Family History | 0.476 | 0.614 | 124 | 0.775 | 0.440 |
| Time Between the Two Scores | -0.003 | 0.008 | 1173 | -0.334 | 0.739 |
| Time ^2 | 0.000 | 0.000 | 1173 | 0.269 | 0.788 |
| Previous MFQ Score | 0.582 | 0.022 | 1173 | 25.965 | <0.001*** |
| Inpatient Status | -0.680 | 0.465 | 1173 | -1.461 | 0.144 |
| Age | -0.177 | 0.111 | 1173 | -1.601 | 0.110 |
| Sex (Male) | -1.294 | 0.445 | 124 | -2.907 | 0.004** |
| Antidepressants at Baseline | -0.007 | 0.446 | 124 | -0.016 | 0.987 |
| Other Medications at Baseline | 0.392 | 0.495 | 124 | 0.792 | 0.430 |
| Post Pandemic | -0.308 | 0.323 | 1173 | -0.952 | 0.341 |
| Interaction between family history and time between | 0.005 | 0.009 | 1173 | 0.492 | 0.623 |
| Interaction between family history and time^2 | 0.000 | 0.000 | 1173 | -0.700 | 0.484 |
| **Model Statistics** | | | | | |
| **AIC** | | **BIC** | | **logLik** | |
| 7639.11 | | 7726.964 | | -3802.556 | |

| **MFQ Analyses** | | | | | |
| --- | --- | --- | --- | --- | --- |
| **Table S12: Linear Mixed Effect Results for MFQ0 Model**  MFQ Score ~ Time Between + Baseline MFQ + Time Between* Baseline MFQ + Antidepressants at Baseline + Other Meds at Baseline + Inpatient + Sex + Age + Post Pandemic, random = Time Between \| Participant ID | | | | | |
| **Variable** | **Estimate** | **Std. Error** | **DF** | **t-Value** | **p-Value** |
| Antidepressants at Baseline | 0.668 | 0.832 | 124 | 0.802 | 0.424 |
| Time Between the two scores | 0.001 | 0.005 | 1176 | 0.254 | 0.800 |
| Baseline MFQ Score | 0.486 | 0.059 | 124 | 8.179 | <0.001*** |
| Inpatient | -0.396 | 0.518 | 1176 | -0.765 | 0.445 |
| Age | -0.449 | 0.179 | 1176 | -2.509 | 0.012* |
| Sex (Male) | -3.315 | 0.823 | 124 | -4.026 | <0.001*** |
| Other Meds at Baseline | -0.531 | 0.934 | 124 | -0.568 | 0.571 |
| Post Pandemic | -1.186 | 0.402 | 1176 | -2.947 | 0.003** |
| Interaction of Time Between Scores and Baseline MFQ Score | 0.000 | 0.000 | 1176 | -1.224 | 0.221 |
| **Model Statistics** | | | | | |
| **AIC** | | **BIC** | | **logLik** | |
| 7907.502 | | 7979.884 | | -3939.751 | |

| **MFQ Analyses** | | | | | |
| --- | --- | --- | --- | --- | --- |
| **Table S13: Linear Mixed Effects Results for MFQ0 + FH Model**  MFQ Score ~ Antidepressants at Baseline + Time Between*Baseline MFQ + Family History + Family History:Time Between + Other Meds at Baseline + Inpatient + Sex + Age + Post Pandemic, random = Time Between \| Participant ID | | | | | |
| **Variable** | **Estimate** | **Std. Error** | **DF** | **t-Value** | **p-Value** |
| Antidepressants at Baseline | 0.635 | 0.836 | 123 | 0.760 | 0.449 |
| Time Between the Two Scores | 0.001 | 0.006 | 1175 | 0.183 | 0.855 |
| Baseline MFQ Score | 0.481 | 0.060 | 123 | 8.035 | <0.001*** |
| Family History | 0.589 | 0.914 | 123 | 0.645 | 0.520 |
| Inpatient Status | -0.396 | 0.519 | 1175 | -0.764 | 0.445 |
| Age | -0.457 | 0.179 | 1175 | -2.546 | 0.011* |
| Sex (Male) | -3.279 | 0.827 | 123 | -3.964 | <0.001*** |
| Other Medications at Baseline | -0.527 | 0.936 | 123 | -0.562 | 0.575 |
| Post Pandemic | -1.176 | 0.403 | 1175 | -2.914 | 0.004 |
| Interaction between time between and baseline MFQ | 0.000 | 0.000 | 1175 | -1.191 | 0.234 |
| Interaction between time between and family history | 0.000 | 0.005 | 1175 | -0.015 | 0.988 |
| **Model Statistics** | | | | | |
| **AIC** | | **BIC** | | **logLik** | |
| 7918.207 | | 8000.904 | | -3943.103 | |

| **MFQ Analyses** | | | | | |
| --- | --- | --- | --- | --- | --- |
| **Table S14: Linear Mixed Effect Results for MFQ0 + MFQ Model**  MFQ Score ~ Time Between* Baseline MFQ + Previous MFQ + Antidepressants at Baseline + Other Meds at Baseline + Inpatient + Sex + Age + Post Pandemic, random = Time Between \| Participant ID | | | | | |
| **Variable** | **Estimate** | **Std. Error** | **DF** | **t-Value** | **p-Value** |
| Antidepressants at Baseline | 0.476 | 0.435 | 124 | 1.092 | 0.277 |
| Time Between the two scores | 0.001 | 0.004 | 1175 | 0.365 | 0.715 |
| Baseline MFQ Score | 0.193 | 0.036 | 124 | 5.307 | <0.001*** |
| Previous MFQ Score | 0.539 | 0.024 | 1175 | 22.777 | <0.001*** |
| Inpatient | -0.835 | 0.441 | 1175 | -1.893 | 0.059 |
| Age | -0.154 | 0.107 | 1175 | -1.448 | 0.148 |
| Sex (Male) | -1.571 | 0.429 | 124 | -3.665 | <0.001*** |
| Other Medications at Baseline | -0.064 | 0.484 | 124 | -0.132 | 0.895 |
| Post Pandemic | -0.448 | 0.314 | 1175 | -1.423 | 0.155 |
| Interaction of Time Between Scores and Baseline MFQ Score | 0.000 | 0.000 | 1175 | -0.999 | 0.318 |
| **Model Statistics** | | | | | |
| **AIC** | | **BIC** | | **logLik** | |
| 7576.857 | | 7654.397 | | -3773.428 | |

| **Table S15: Demographic Characteristics of Sample** | |
| --- | --- |
| **Variable** | **Baseline Weeks of Depression Dataset** |
| N | 92 People |
| Mean Age | 15.79 (SD= 1.39) |
| % Female | 72% |
| % with a Positive Family History of Depression in a First Degree Relative | 74% |
| Mean MFQ Score at Baseline | 12.38 (SD= 6.84) |
| % Taking an Antidepressant at the Baseline Visit | 43% |
| % Taking another Psychiatric Medication at the Baseline Visit | 29% |
| Requirements for Inclusion | -MDD or s-MDD Participant  -Family History Interview Complete  -Baseline MFQ Score Present  -Value for Weeks of Depression Collected at the 1 Year Follow Up  -Medication Data from Baseline |

| **Weeks of Depression Analyses** | | | | |
| --- | --- | --- | --- | --- |
| **Table S16: Linear Regression Results for Baseline Null Model**  Weeks of Depression ~ Antidepressants at Baseline + Other Meds at Baseline + Sex + Age at Baseline + Post Pandemic | | | | |
| **Variable** | **Estimate** | **Std. Error** | **t-Value** | **p-Value** |
| Antidepressants at Baseline | 3.732 | 4.564 | 0.818 | 0.416 |
| Other Meds at Baseline | -5.145 | 4.942 | -1.041 | 0.301 |
| Sex (Male) | 0.147 | 4.371 | 0.034 | 0.973 |
| Age | 1.428 | 1.429 | 1.000 | 0.320 |
| Post Pandemic | 6.607 | 4.043 | 1.634 | 0.106 |
| **Model Statistics** | | | | |
| **Residual Standard Error** | **Multiple R Squared** | **Adjusted R Squared** | **F Statistic** | **Model p-Value** |
| 18.74 on 86 df | 0.051 | -0.004 | 0.923 on 5 and 86 df | 0.470 |

| **Weeks of Depression Analyses** | | | | |
| --- | --- | --- | --- | --- |
| **Table S17: Linear Regression Results for Baseline FH Model**  Weeks of Depression ~ Family History + Antidepressants at Baseline + Other Meds at Baseline + Sex + Age at Baseline + Post Pandemic | | | | |
| **Variable** | **Estimate** | **Std. Error** | **t-Value** | **p-Value** |
| Family History | 8.319 | 4.488 | 1.854 | 0.067 |
| Antidepressants at Baseline | 3.051 | 4.516 | 0.676 | 0.501 |
| Other Meds at Baseline | -4.768 | 4.877 | -0.978 | 0.331 |
| Sex (Male) | 0.104 | 4.311 | 0.024 | 0.981 |
| Age | 0.909 | 1.436 | 0.633 | 0.528 |
| Post Pandemic | 6.269 | 3.991 | 1.571 | 0.120 |
| **Model Statistics** | | | | |
| **Residual Standard Error** | **Multiple R Squared** | **Adjusted R Squared** | **F Statistic** | **Model p-Value** |
| 18.48 on 85 df | 0.088 | 0.023 | 1.363 on 6 and 85 df | 0.239 |

| **Weeks of Depression Analyses** | | | | |
| --- | --- | --- | --- | --- |
| **Table S18: Linear Regression Results for Baseline MFQ Model**  Weeks of Depression ~ Baseline MFQ + Antidepressants at Baseline + Other Meds at Baseline + Sex + Age at Baseline + Post Pandemic | | | | |
| **Variable** | **Estimate** | **Std. Error** | **t-Value** | **p-Value** |
| Baseline MFQ | 1.113 | 0.275 | 4.051 | <0.001*** |
| Antidepressants at Baseline | 6.731 | 4.268 | 1.577 | 0.118 |
| Other Meds at Baseline | -7.980 | 4.604 | -1.733 | 0.087 |
| Sex (Male) | -0.977 | 4.035 | -0.242 | 0.809 |
| Age | 0.503 | 1.335 | 0.377 | 0.707 |
| Post Pandemic | 5.392 | 3.735 | 1.444 | 0.152 |
| **Model Statistics** | | | | |
| **Residual Standard Error** | **Multiple R Squared** | **Adjusted R Squared** | **F Statistic** | **Model p-Value** |
| 17.25 on 85 df | 0.205 | 0.148 | 3.643 on 6 and 85 df | 0.003** |

| **Weeks of Depression Analyses** | | | | |
| --- | --- | --- | --- | --- |
| **Table S19: Linear Regression Results for Baseline MFQ + FH Model**  Weeks of Depression ~ Baseline MFQ + Family History + Antidepressants at Baseline + Other Meds at Baseline + Sex + Age at Baseline + Post Pandemic | | | | |
| **Variable** | **Estimate** | **Std. Error** | **t-Value** | **p-Value** |
| Family History | 8.504 | 4.113 | 2.068 | 0.042* |
| Baseline MFQ | 1.119 | 0.270 | 4.151 | <0.001* |
| Antidepressants at Baseline | 6.050 | 4.201 | 1.440 | 0.154 |
| Other Meds at Baseline | -7.611 | 4.521 | -1.683 | 0.096 |
| Sex (Male) | -1.027 | 3.959 | -0.259 | 0.796 |
| Age | -0.032 | 1.336 | -0.024 | 0.981 |
| Post Pandemic | 5.040 | 3.669 | 1.374 | 0.173 |
| **Model Statistics** | | | | |
| **Residual Standard Error** | **Multiple R Squared** | **Adjusted R Squared** | **F Statistic** | **Model p-Value** |
| 16.93 on 84 df | 0.243 | 0.18 | 3.853 on 7 and 84 df | 0.001** |

| **Table S20: Demographic Characteristics of Sample for Parent Report sensitivity analysis** | | |
| --- | --- | --- |
| **Variable** | **Dataset for Question 2 (Depressive Severity, Self-Report MFQ)** | **Dataset for Question 2 with Parent Report (Depressive Severity, Parent Report MFQ)** |
| N | 129 | 122 |
| Number of assessments | 1439 | 925 |
| Mean number of assessments | 11.15 (SD = 6.71) | 7.58 (SD =5.23) |
| Interval between assessments (Days) | Median = 36.5 (IQR = 96) | Median = 58 (IQR = 111) |
| Mean Age (Years) | 15.61 (SD = 1.50) | 15.50 (SD = 1.50) |
| % Female | 75% | 75% |
| % with a Positive FHD in a First Degree Relative | 74% | 73% |
| Mean MFQ Score | 10.95 (SD= 5.32) (Across all people and timepoints) | 6.76 (SD= 5.57) (Across all people and timepoints) |
| % Taking an Antidepressant at the Baseline Visit | 43% | 43% |
| % Taking another Psychiatric Medication at the Baseline Visit | 26% | 27% |
| Requirements for Inclusion | -MDD or s-MDD Participant  -Family History Interview Complete  -At Least 2 self-report MFQ Scores Present  -Medication Data from Baseline | -MDD or s-MDD Participant  -Family History Interview Complete  -At Least 2 parent MFQ Scores Present  -Medication Data from Baseline |

| **Table S21: Demographic Characteristics of Sample for Sensitivity Analysis of Different Intervisit Interval Subsets for the analysis of Depressive Severity** | | | | |
| --- | --- | --- | --- | --- |
| **Variable** | **Short Interval (1-15 days)** | **Medium Interval (16-77 days)** | **Long Interval (78-518 days)** | **First & Last** |
| N | 87 | 114 | 121 | 129 |
| Number of assessments | 441 | 425 | 444 | 129 |
| Mean number of assessments | 5.07(SD =4.92) | 3.73 (SD =2.05) | 3.67 (SD = 1.84) | 1 |
| Median intervisit interval (Days) | 7 (IQR = 6) | 36 (IQR = 34) | 127 (IQR = 67) | 727 (IQR = 588) |
| Mean Age (Years) | 16.34 (SD = 1.91) | 16.25 (SD = 1.76) | 15.69 (SD = 1.52) | 15.61 (SD = 1.50) |
| % Female | 72% | 76% | 75% | 75% |
| % FHD | 74% | 72% | 74% | 74% |
| Grand Mean MFQ Score | 11.80 (SD= 6.69) | 10.36 (SD= 6.14) | 10.02 (SD = 6.45) | 11.14 (SD = 7.07) |
| % Taking an Antidepressant at the Baseline Visit | 43% | 45% | 44% | 43% |
| % Taking another Psychiatric Medication at the Baseline Visit | 26% | 26% | 26% | 26% |

**Supplemental Figures**


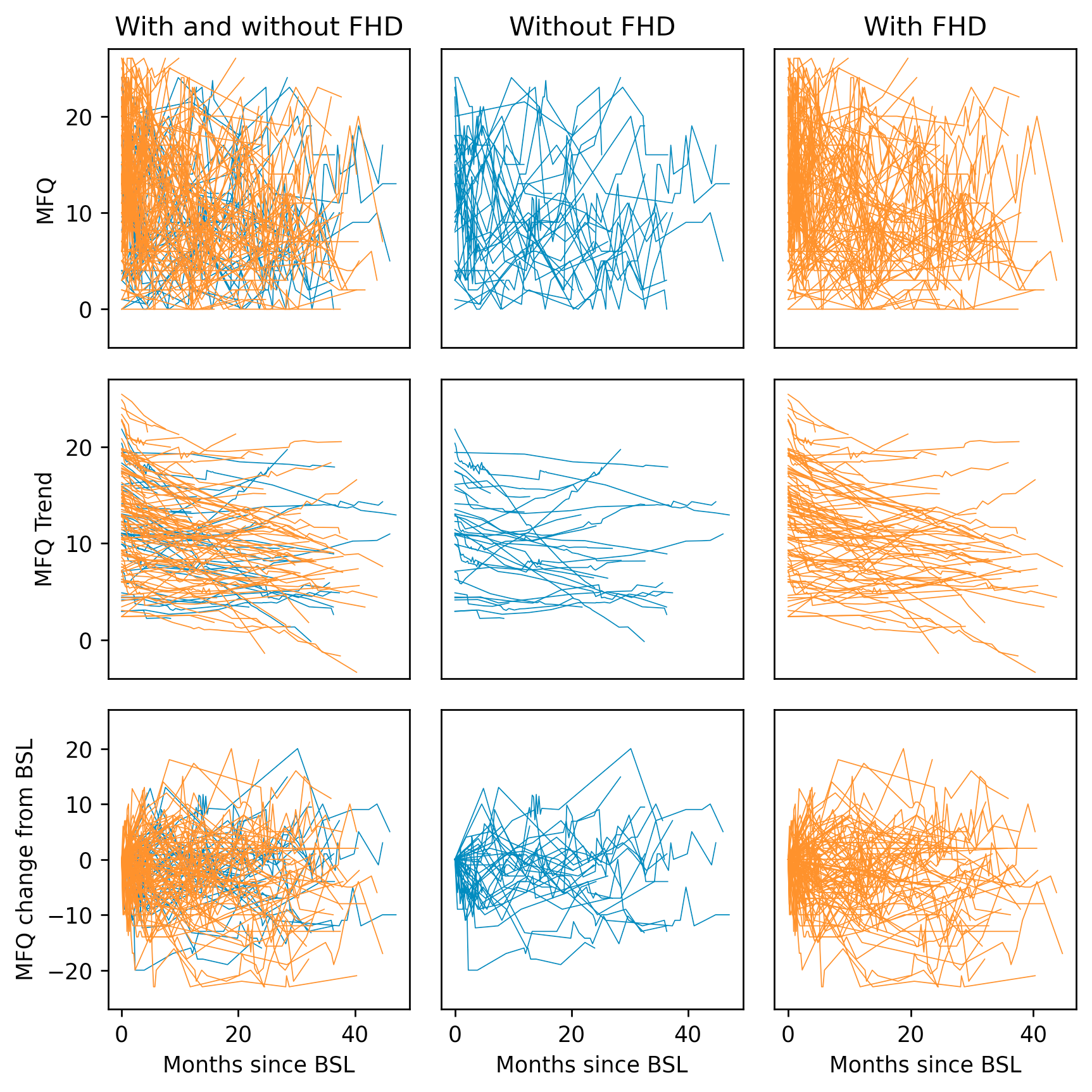


**Figure S1:** Spaghetti plots of all of the MFQ trajectories. The first column shows individuals with and without an FHD together, the second column just those without an FHD, the third column just those with an FHD. The first row shows raw MFQ scores. The second row shows MFQ scores predicted by a mixed effects model fit to the data in order to smooth out some of the noise in the raw scores and give a sense of the overall trajectories. The final row shows the change in MFQ score from baseline as another way to give a sense of trajectories over time.

**
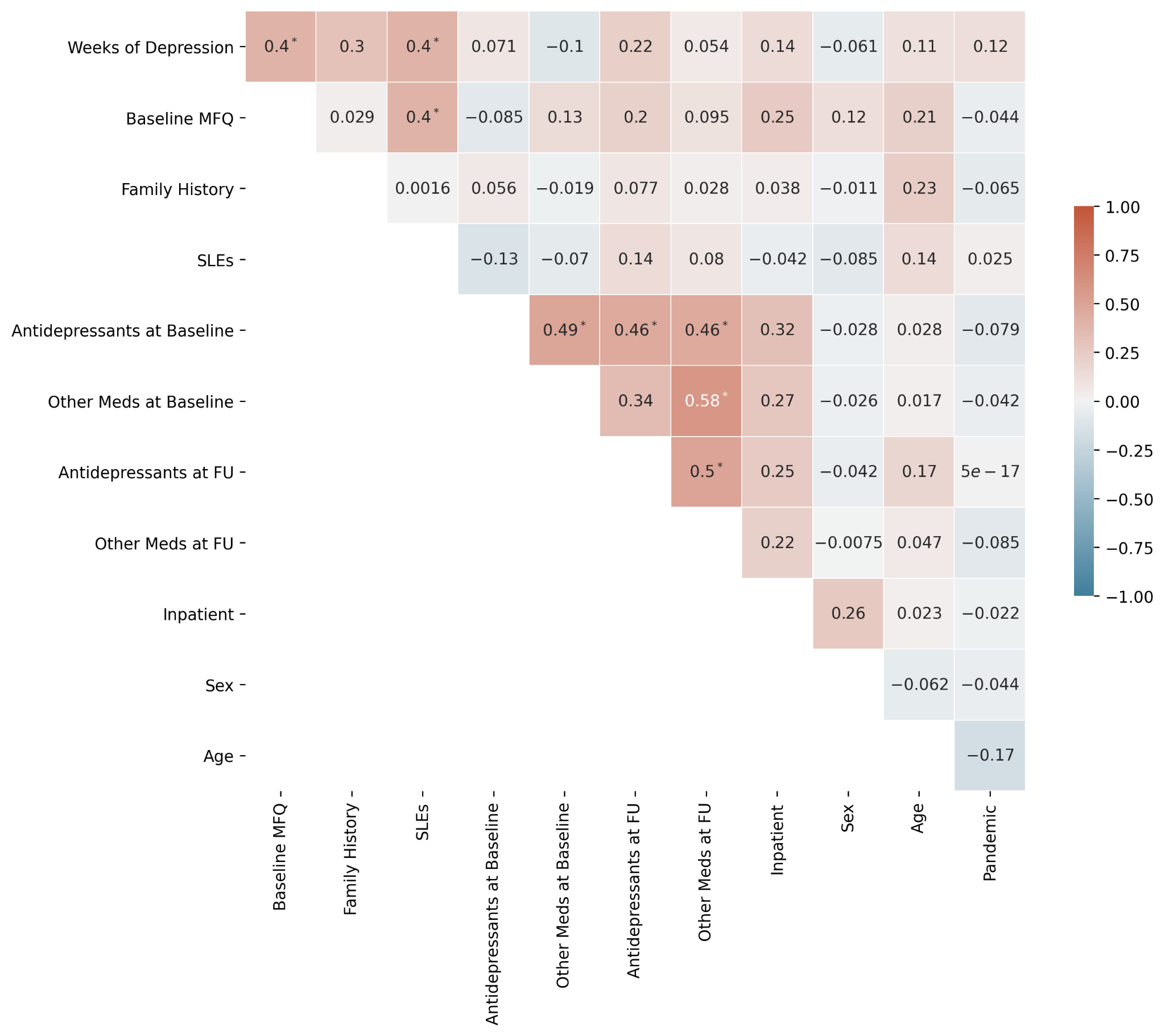
**

**Figure S2:** Pairwise Pearson correlations between terms in the Weeks of Depression analysis. *: significant correlation after correcting for multiple comparisons.

**
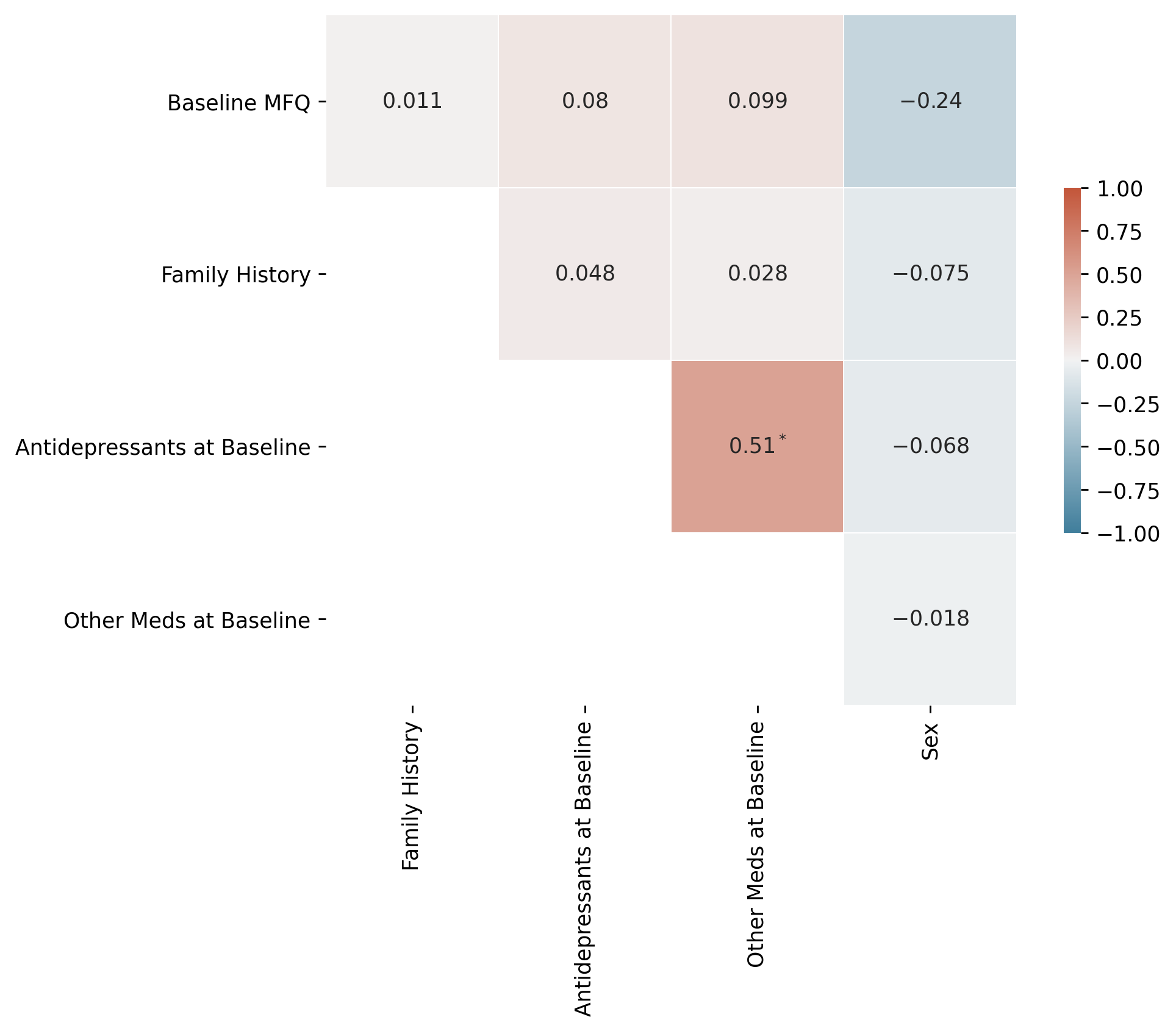
**

**Figure S3:** Pairwise Pearson correlations between terms with a single value per participant in the MFQ analysis. *: significant correlation after correcting for multiple comparisons across Figure S3 and Figure S4.


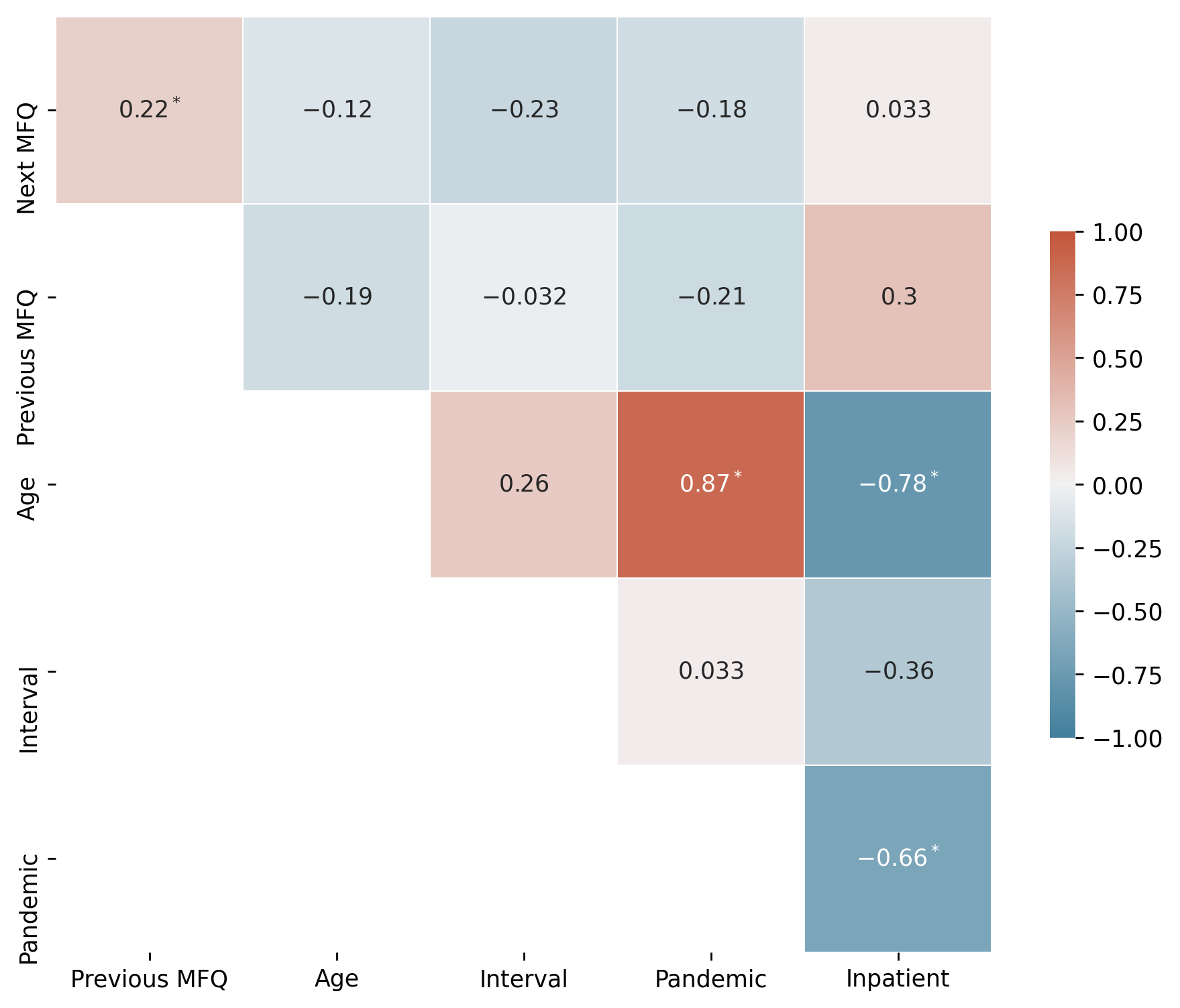


**Figure S4:** Pairwise Pearson correlations between terms with a unique value per pair of visits in the MFQ analysis. *: significant correlation after correcting for multiple comparisons across Figure S3 and Figure S4.

**
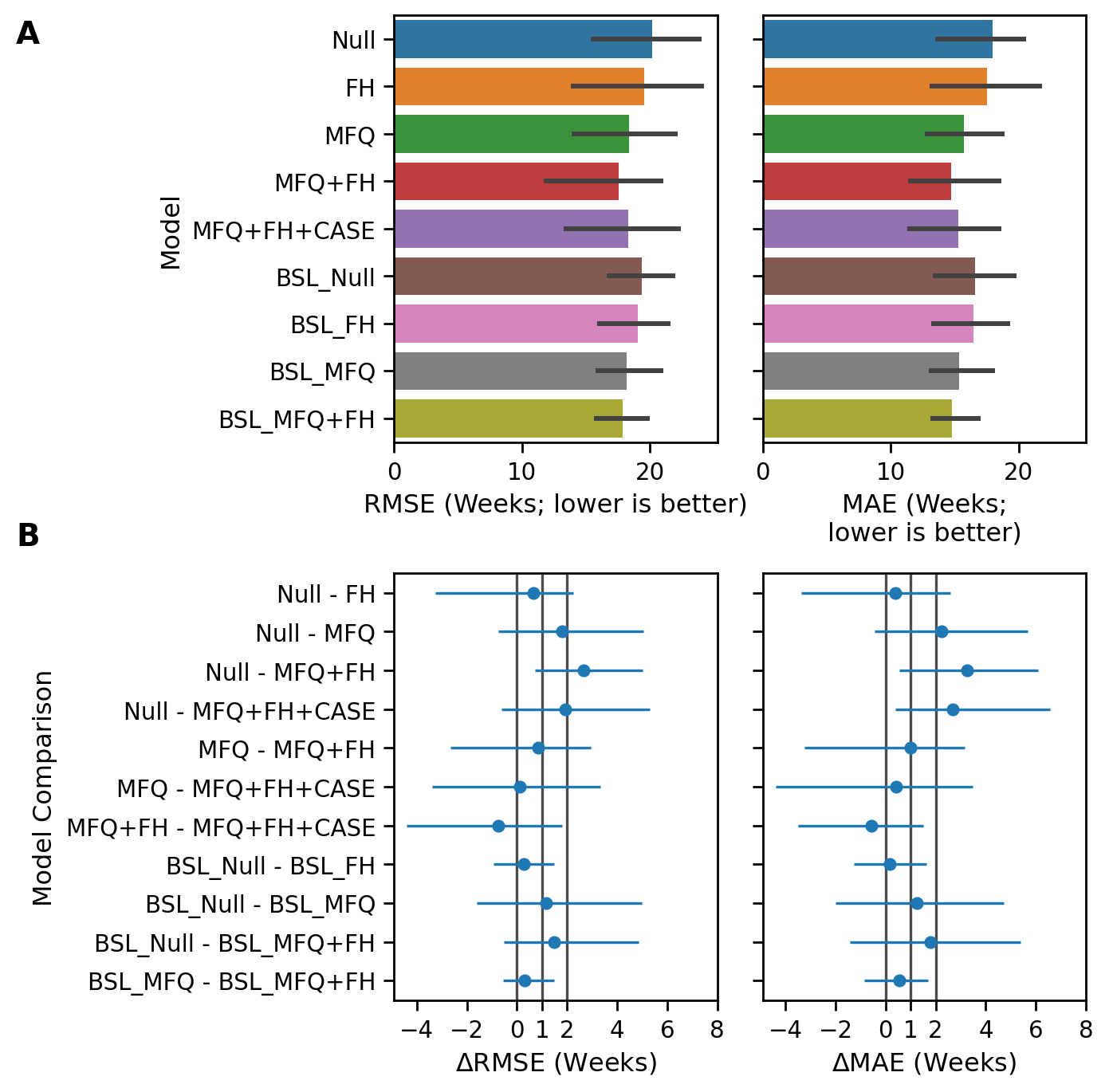
**

**Figure S5:** These are the expanded results for prediction of weeks of depression. The top panel (A) shows the root mean squared error (RMSE) and mean absolute error (MAE) for each model along with bootstrap 99.9% confidence intervals. The lower panel (B) shows comparisons of interest between models in A. Each dot represents the mean difference in RMSE or MAE, while the error bars represent 99.9% confidence intervals. 2 weeks of depression is the defined minimum length for an episode of depression, and half of this value is also shown.

**
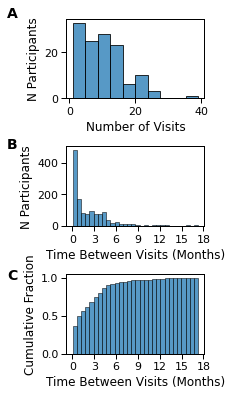
**

**Figure S6:** Distributions of data included in MFQ analysis. The top panel (A) shoes that the majority of participants had fewer than 20 visits included. Most pairs of visits had an interval of less than 3 months (B and C).

**
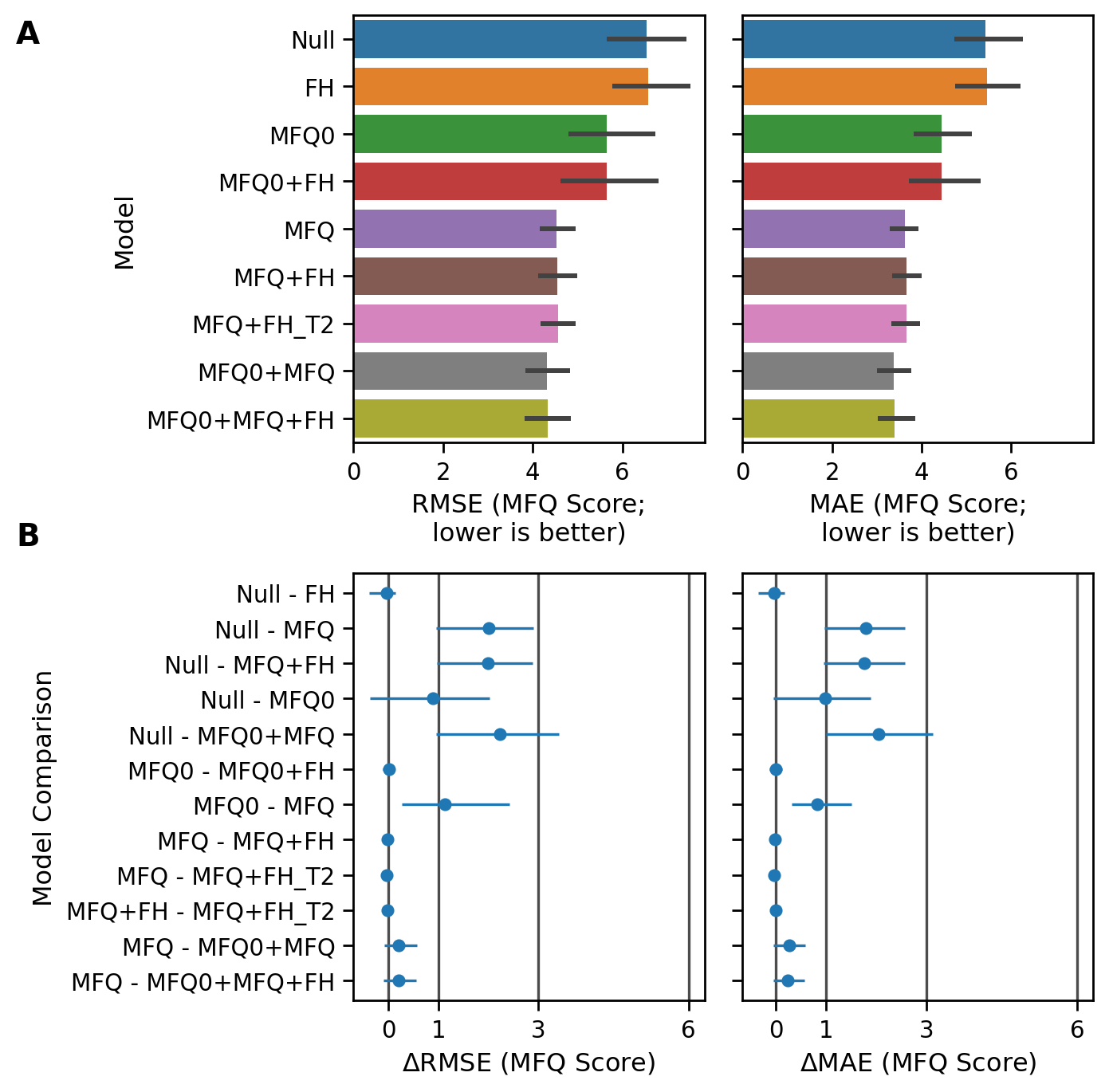
**

**Figure S7:** These are the expanded results for prediction of depression severity as measured by the MFQ. The top panel (A) shows the root mean squared error (RMSE) and mean absolute error (MAE) for each model along with bootstrap 99.9% confidence intervals. The lower panel (B) shows comparisons of interest between models in A. Each dot represents the mean difference in RMSE or MAE, while the error bars represent 99.9% confidence intervals. 6 points on the MFQ is the minimum clinical difference, and half of this value is also shown.


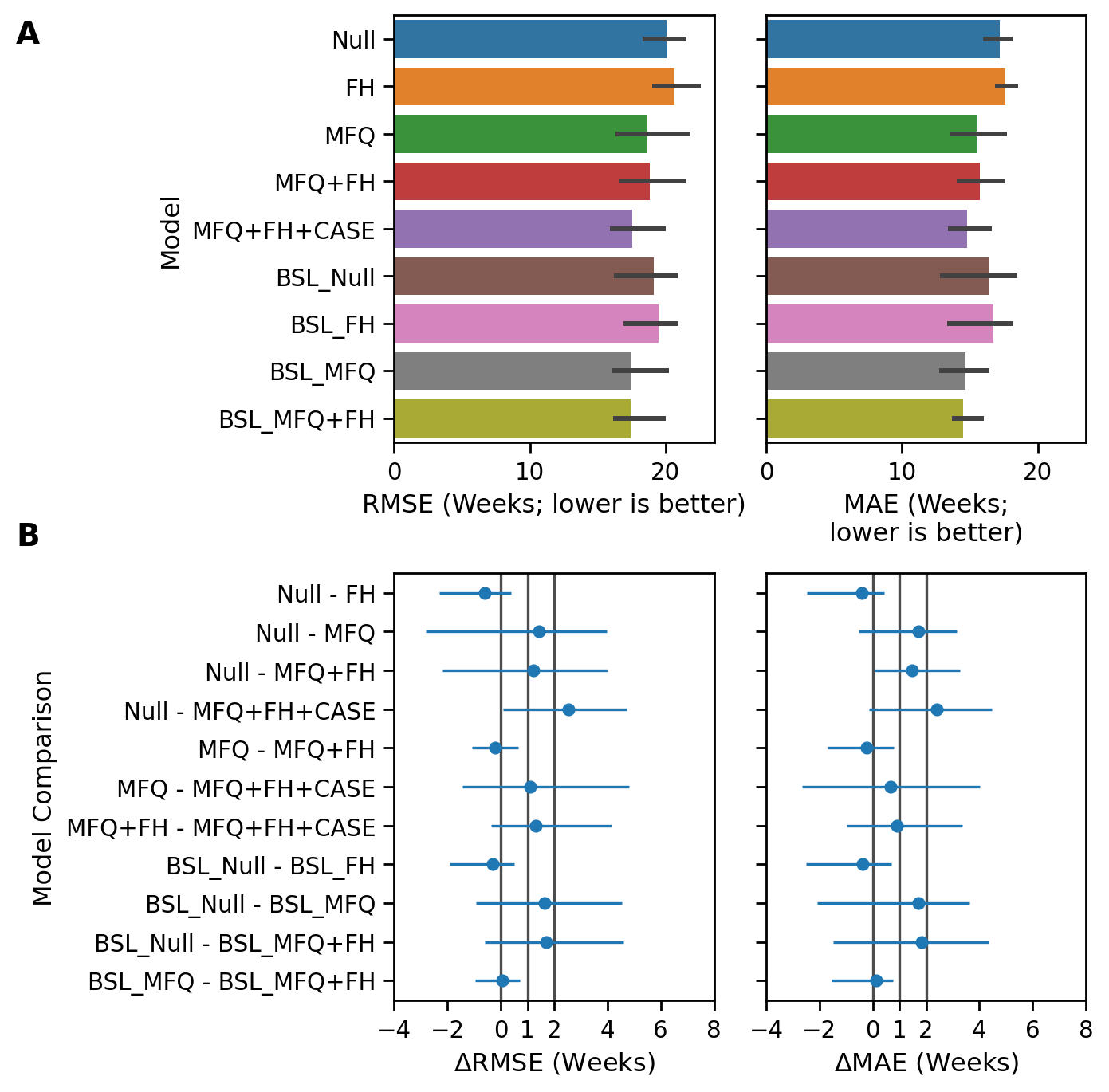


**Figure S8**: This shows the sensitivity analysis using family history of depression diagnosis (as opposed to “some symptoms” and/or diagnosis) as a predictor of weeks of depression. The top panel (A) shows the root mean squared error (RMSE) and mean absolute error (MAE) for each model along with bootstrap 99.9% confidence intervals. The lower panel (B) shows comparisons of interest between models in A. Each dot represents the mean difference in RMSE or MAE, while the error bars represent 99.9% confidence intervals. 2 weeks of depression is the defined minimum length for an episode of depression, and half of this value is also shown. In no cases does Family History of diagnosis improve the MAE or RMSE by more than 2 weeks (Null - MFQ, and MFQ - MFQ+FH).

**
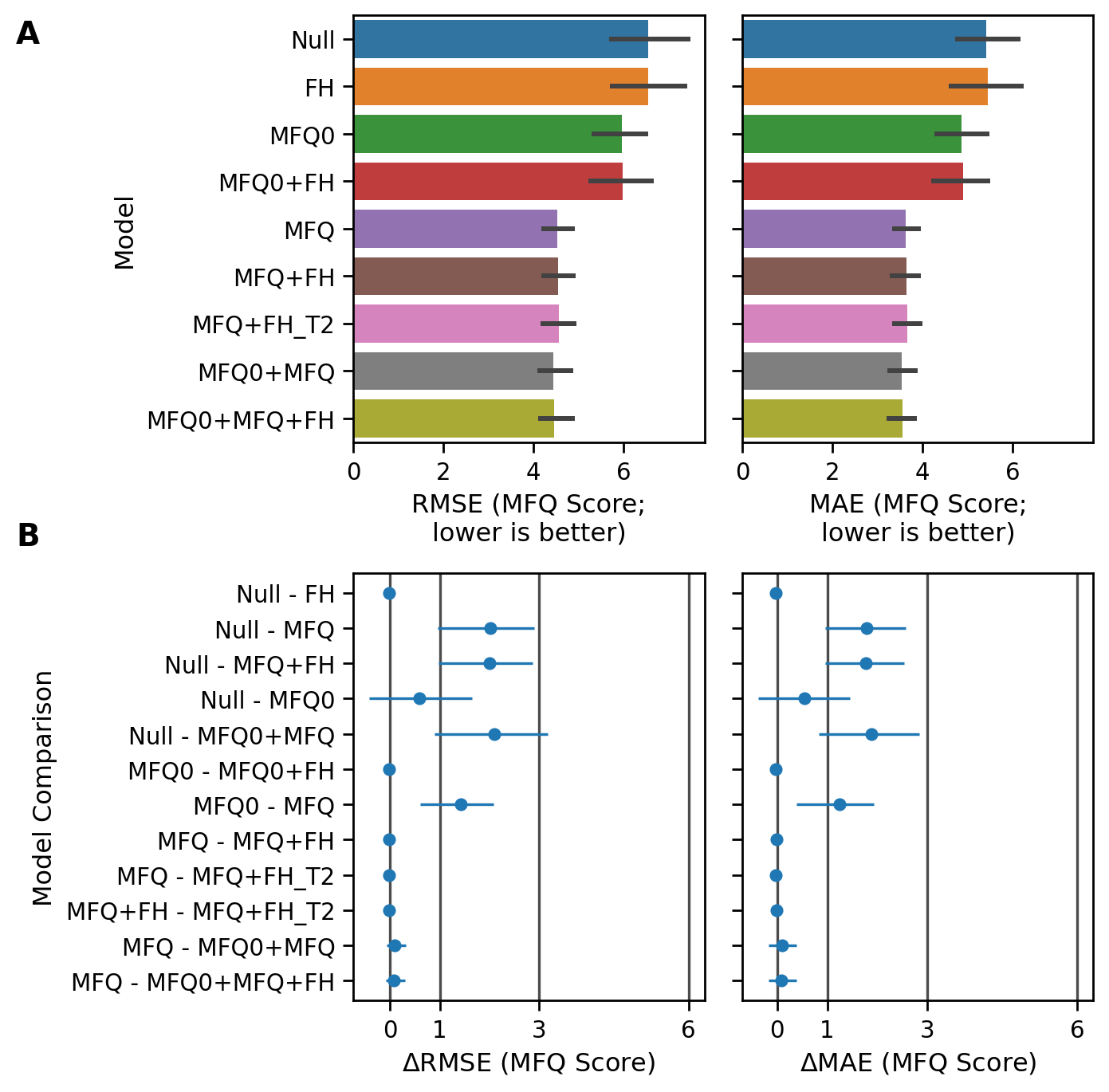
**

**Figure S9**: This shows the sensitivity analysis using family history of depression diagnosis (as opposed to “some symptoms” and/or diagnosis) as a predictor of depression severity as measured by MFQ. The top panel (A) shows the root mean squared error (RMSE) and mean absolute error (MAE) for each model along with bootstrap 99.9% confidence intervals. The lower panel (B) shows comparisons of interest between models in A. Each dot represents the mean difference in RMSE or MAE, while the error bars represent 99.9% confidence intervals. 6 points on the MFQ is the minimum clinical difference, and half of this value is also shown.


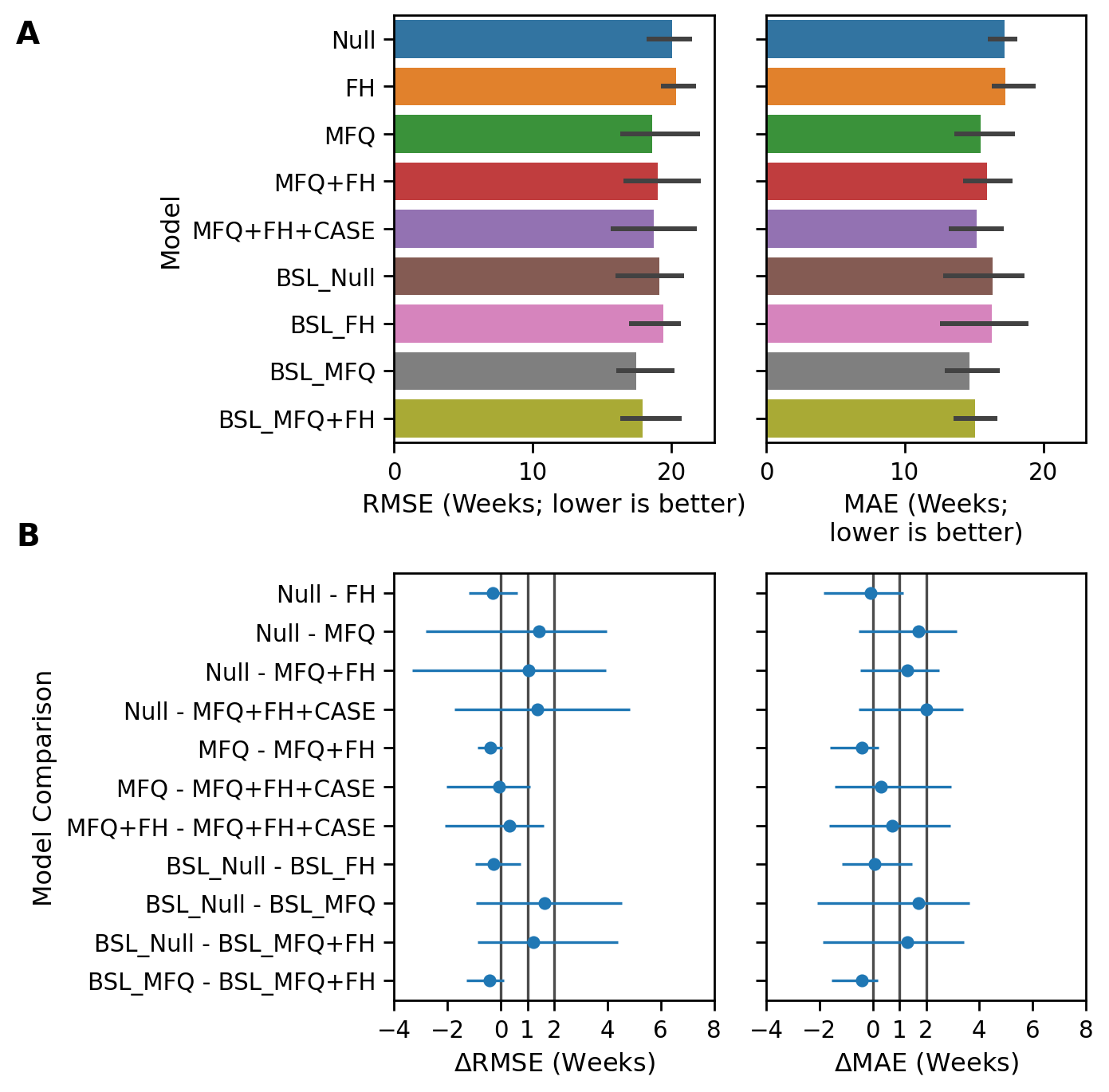


**Figure S10**: This shows the sensitivity analysis using family history of anxiety (FHA) as a predictor of weeks of depression. The top panel (A) shows the root mean squared error (RMSE) and mean absolute error (MAE) for each model along with bootstrap 99.9% confidence intervals. The lower panel (B) shows comparisons of interest between models in A. Each dot represents the mean difference in RMSE or MAE, while the error bars represent 99.9% confidence intervals. 2 weeks of depression is the defined minimum length for an episode of depression, and half of this value is also shown. In no cases does Family History of diagnosis improve the RMSE or MAE by more than 1 week (Null - MFQ, and MFQ - MFQ+FH).

**
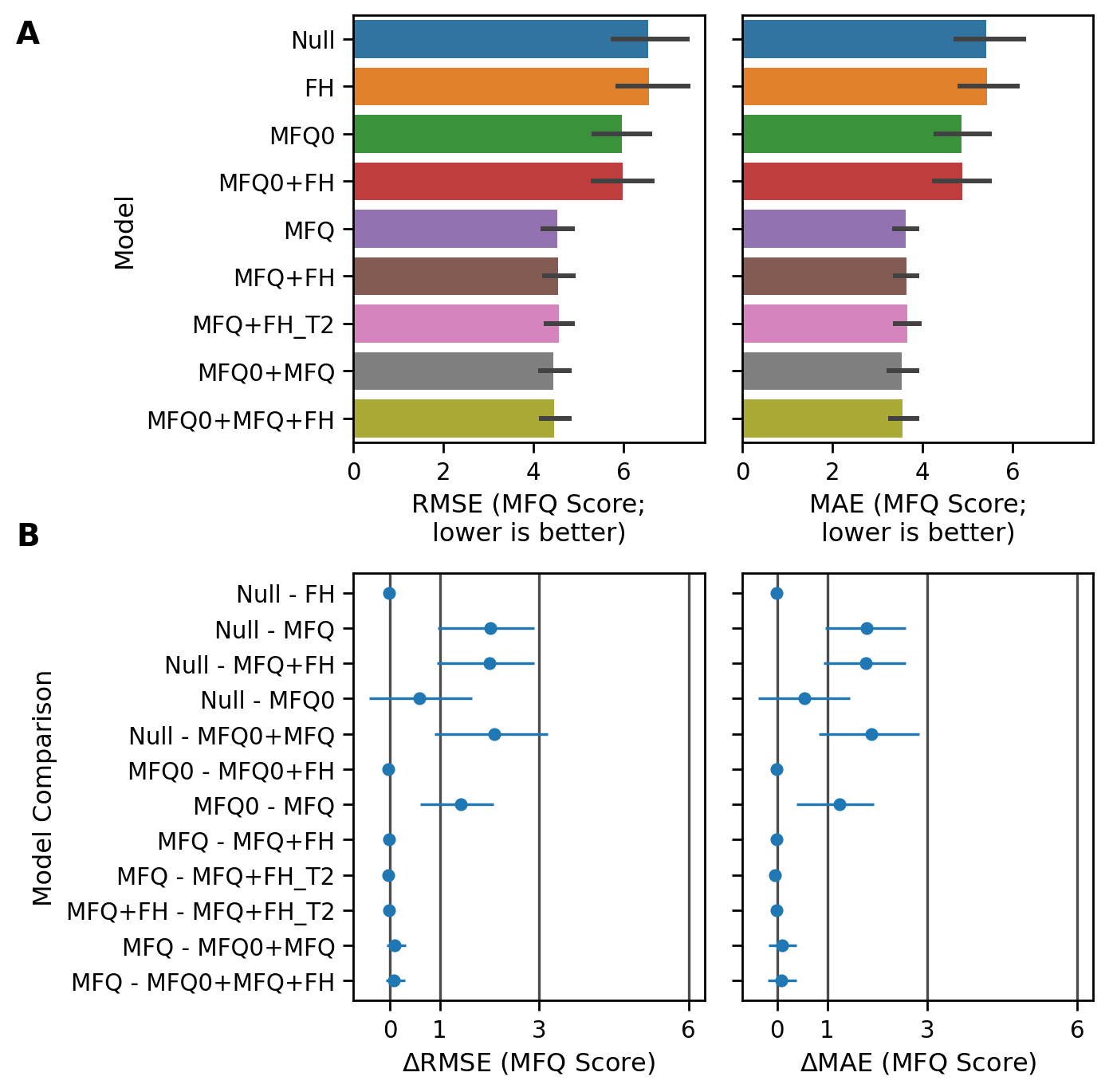
**

**Figure S11**: This shows the sensitivity analysis using family history of anxiety (FHA) as a predictor of depression severity as measured by MFQ. The top panel (A) shows the root mean squared error (RMSE) and mean absolute error (MAE) for each model along with bootstrap 99.9% confidence intervals. The lower panel (B) shows comparisons of interest between models in A. Each dot represents the mean difference in RMSE or MAE, while the error bars represent 99.9% confidence intervals. 6 points on the MFQ is the minimum clinical difference, and half of this value is also shown. In no cases does Family History of diagnosis improve the RMSE or MAE by more than 1 week (Null - MFQ, and MFQ - MFQ+FH).


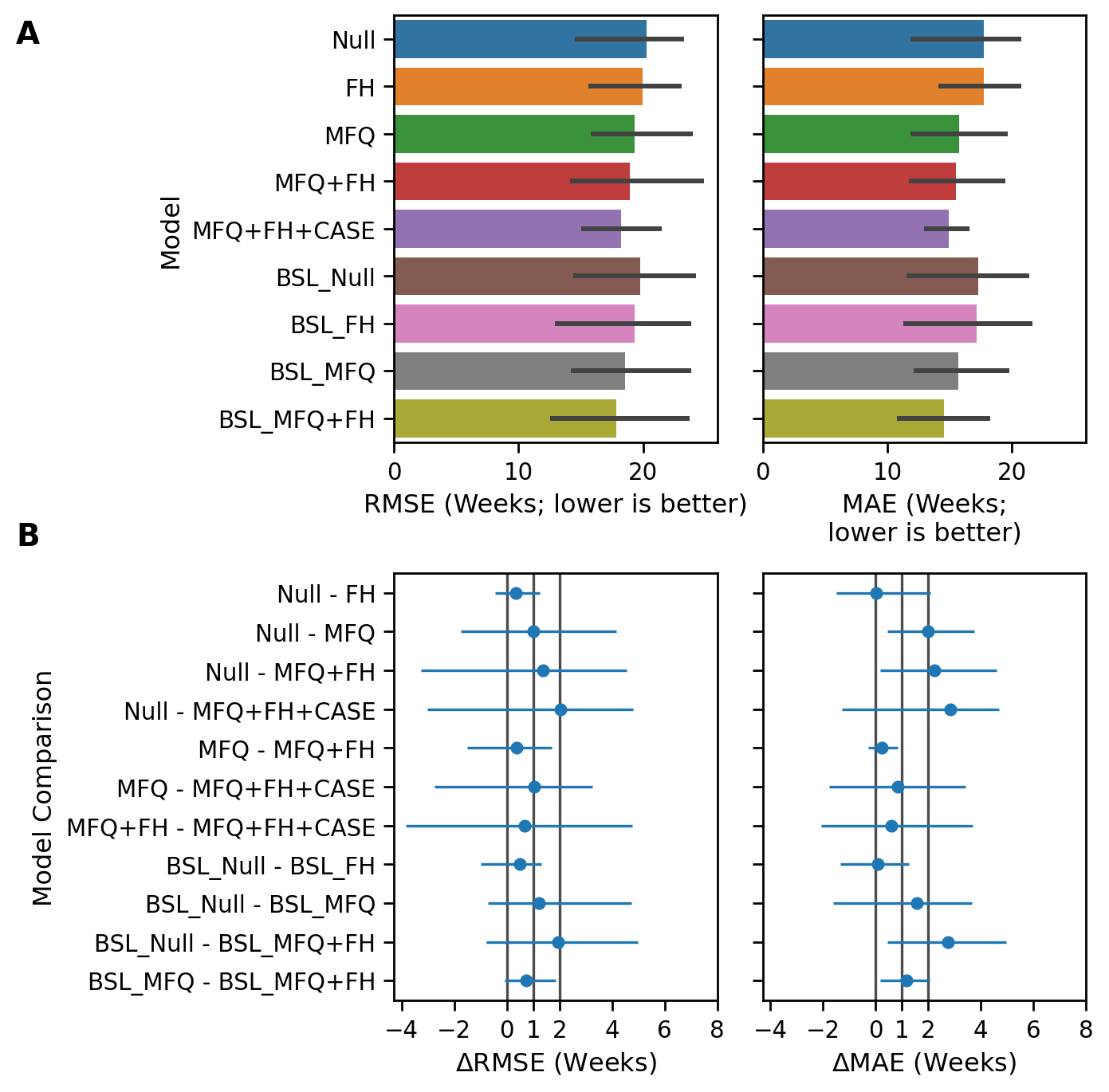


**Figure S12:** This shows the sensitivity analysis excluding all current and former inpatients with family history of depression as a predictor of weeks of depression. The top panel (A) shows the root mean squared error (RMSE) and mean absolute error (MAE) for each model along with bootstrap 99.9% confidence intervals. The lower panel (B) shows comparisons of interest between models in A. Each dot represents the mean difference in RMSE or MAE, while the error bars represent 99.9% confidence intervals. 2 weeks of depression is the defined minimum length for an episode of depression, and half of this value is also shown.

**
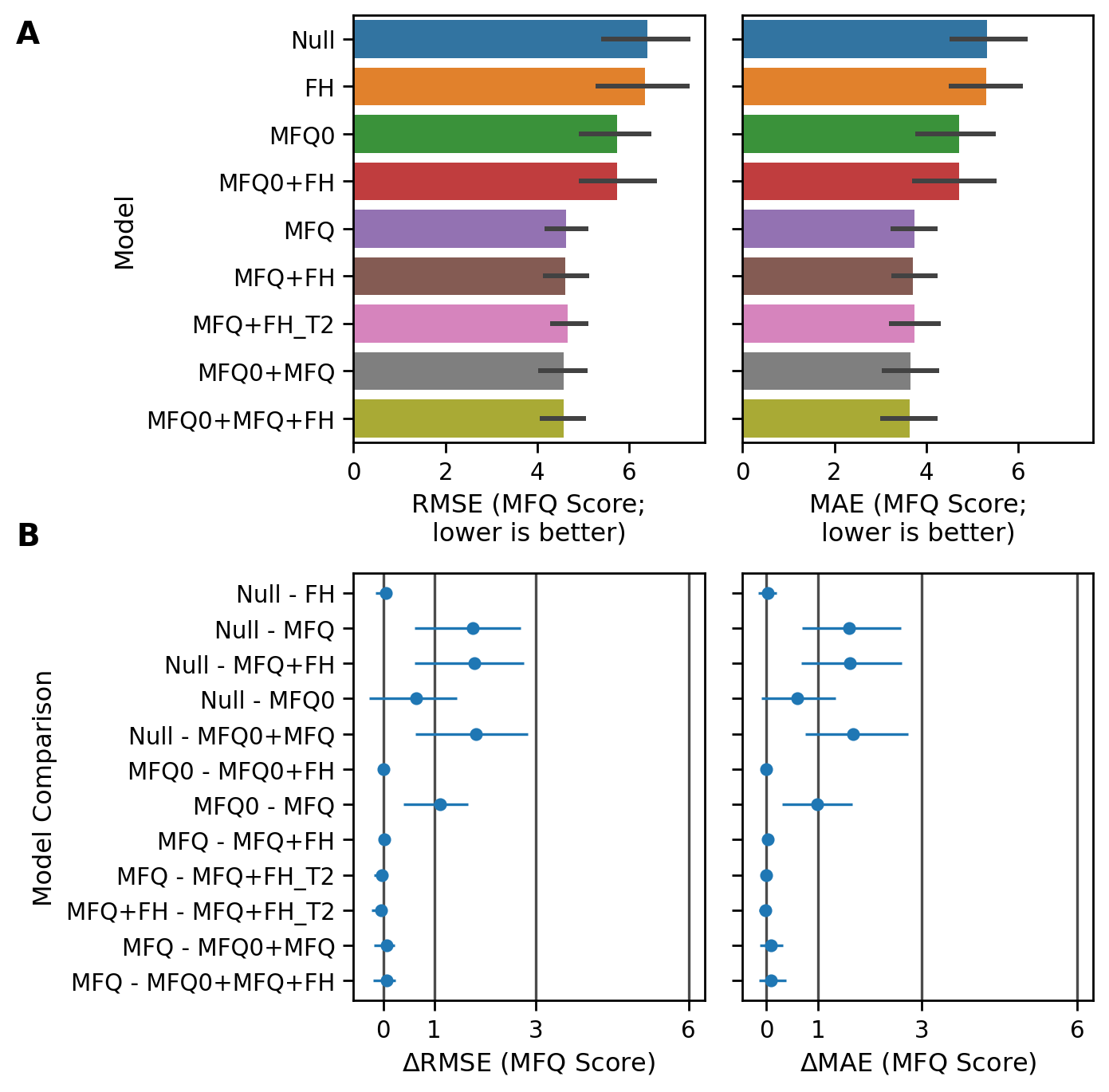
Figure S13:** This shows the sensitivity analysis excluding all current and former inpatients with family history of depression as a predictor of depression severity as measured by the MFQ. The top panel (A) shows the root mean squared error (RMSE) and mean absolute error (MAE) for each model along with bootstrap 99.9% confidence intervals. The lower panel (B) shows comparisons of interest between models in A. Each dot represents the mean difference in RMSE or MAE, while the error bars represent 99.9% confidence intervals. 6 points on the MFQ is the minimum clinical difference, and half of this value is also shown.

**
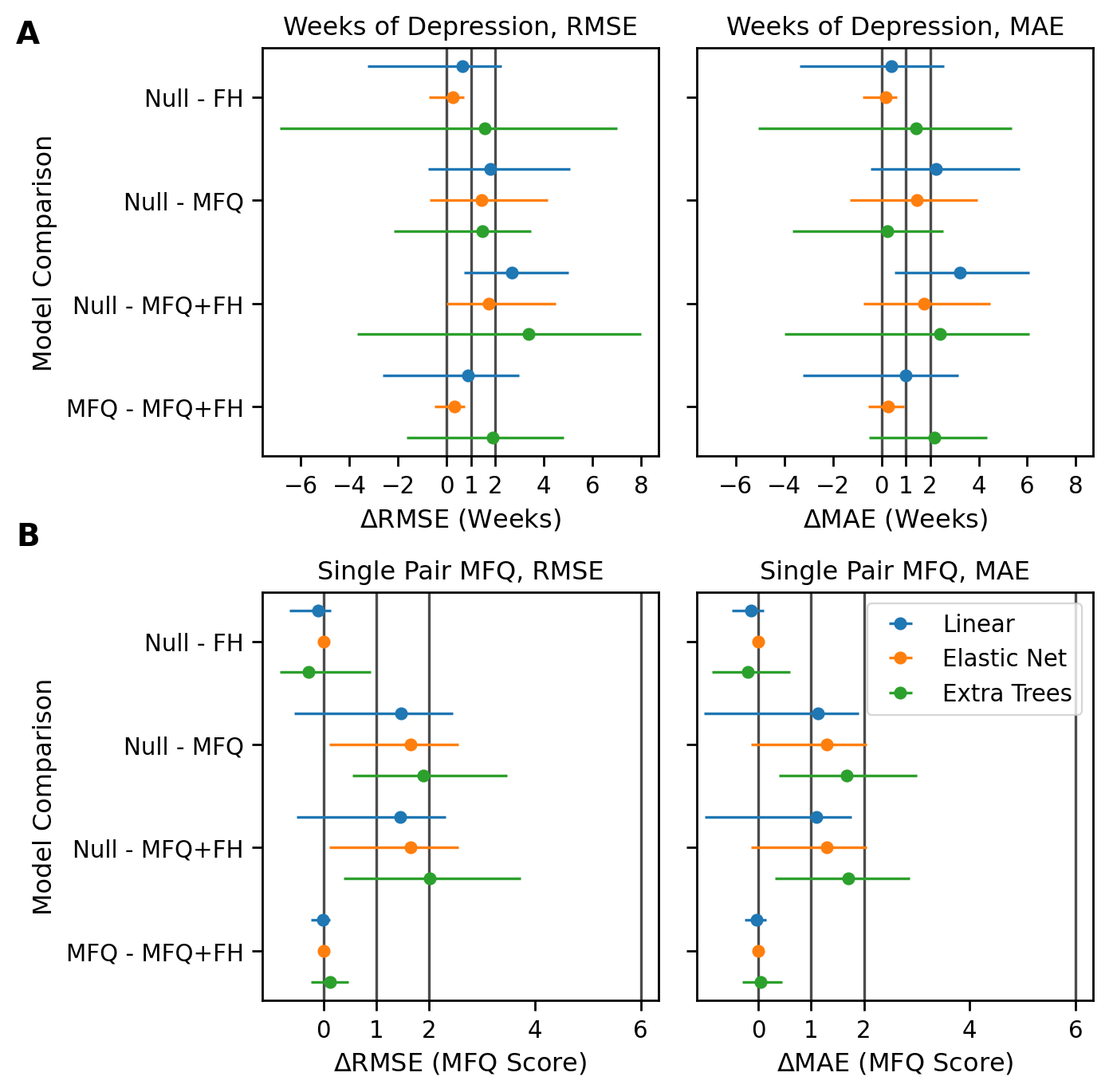
**

**Figure S14:** Comparison of changes in model performance when using Linear Models, Elastic Net, and Extra Trees. Each subplot shows the root mean squared error (RMSE; on the left) and mean absolute error (MAE; on the right) for each model comparison along with bootstrap 99.9% confidence intervals. The top panel (A) shows results for predicting weeks of depression from family history of depression. The lower panel (B) shows results for predicting depression severity as measured by the MFQ from family history of depression. For the MFQ analyses we used just the first pair of visits to avoid issues with nesting of multiple assessments within each participant. In no cases does the addition of family history improve the performance of the models.


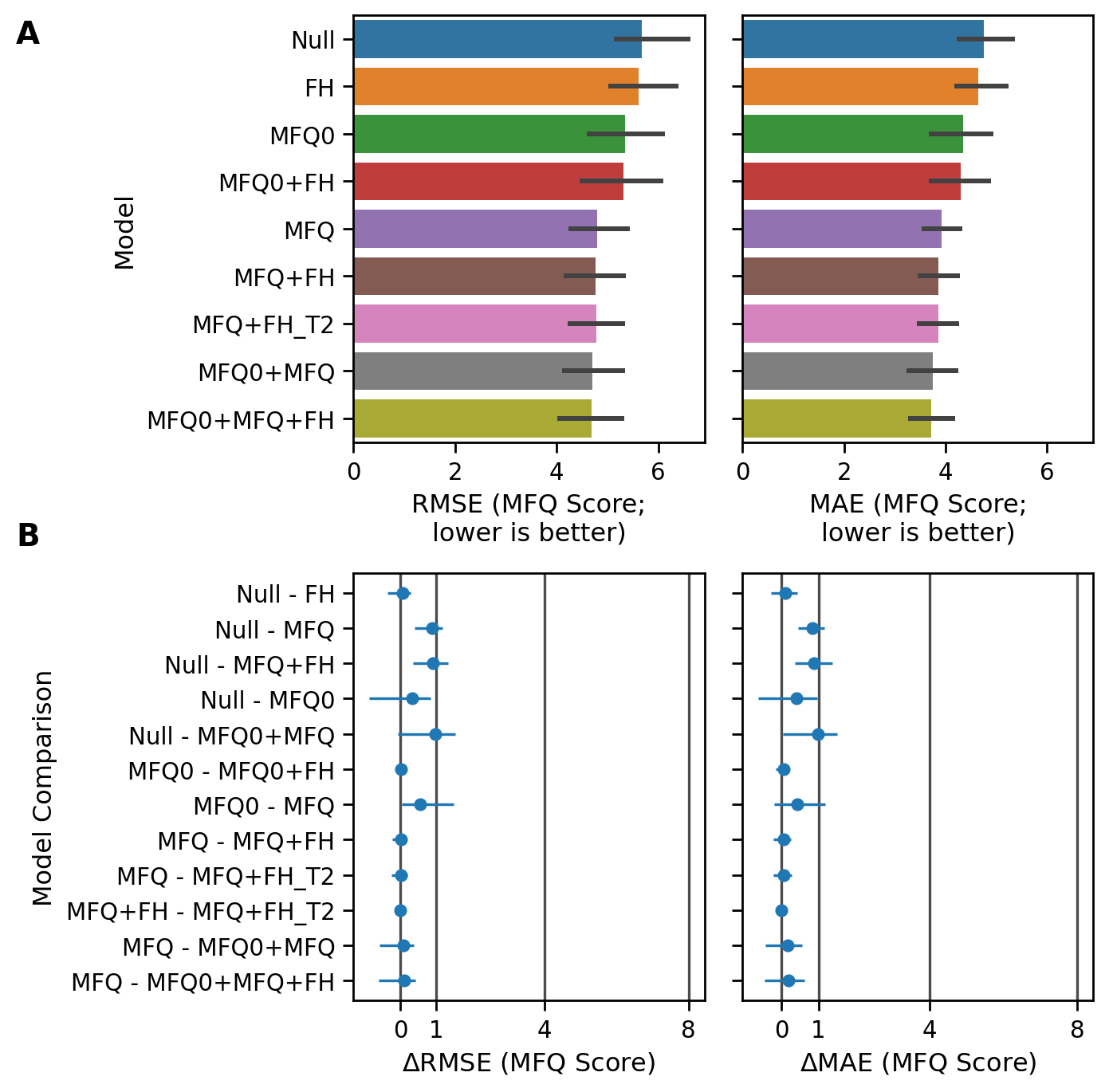


**Figure S15:** This shows the sensitivity analysis with family history of depression as a predictor of depression severity as measured by parent-report MFQ. The top panel (A) shows the root mean squared error (RMSE) and mean absolute error (MAE) for each model along with bootstrap 99.9% confidence intervals. The lower panel (B) shows comparisons of interest between models in A. Each dot represents the mean difference in RMSE or MAE, while the error bars represent 99.9% confidence intervals. 8 points on the parent MFQ is the minimum clinical difference, and half of this value is also shown.

**
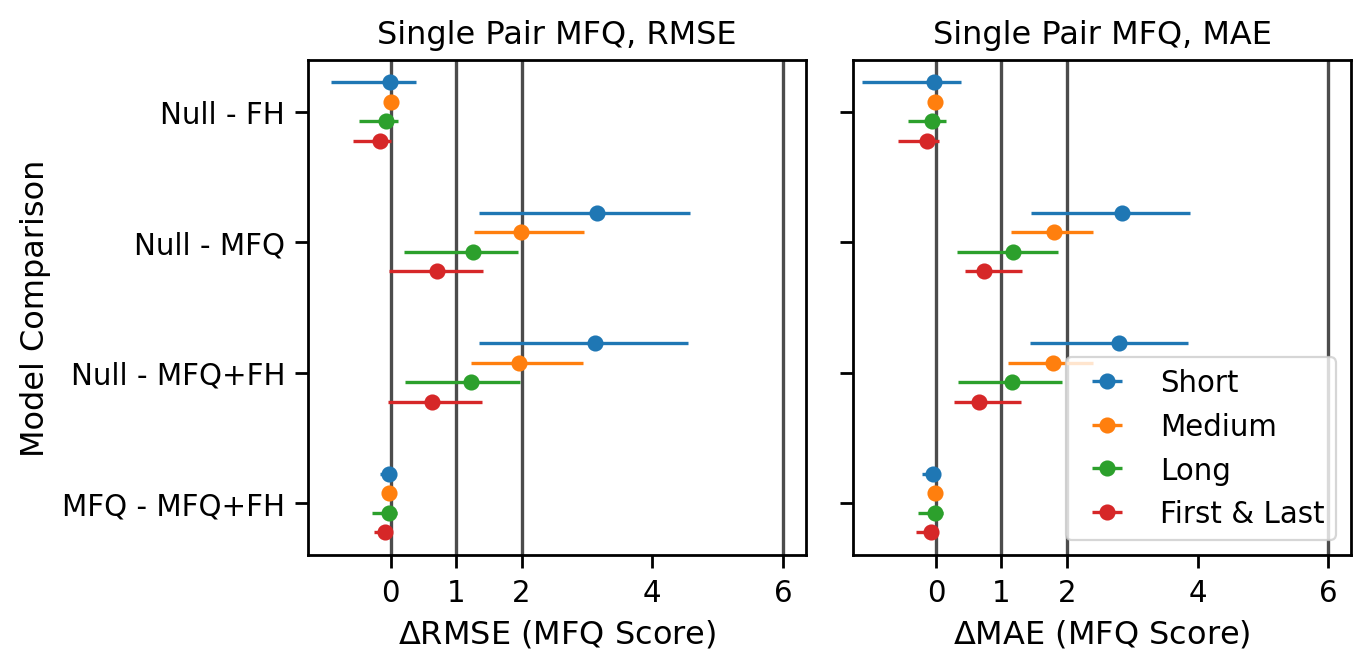
Figure S16:** Comparison of differences in model performance for predicting depression severity as measured by the MFQ from family history of depression when using visits separated by different intervals. Each subplot shows the root mean squared error (RMSE; on the left) and mean absolute error (MAE; on the right) for each model comparison along with bootstrap 99.9% confidence intervals. Short (1-15 days), medium (16-77 days), and long (77-518 days) intervals represent tercile split of the intervisit interval. First & Last represents predicting the final MFQ score from the initial, allowing us to assess the longest intervisit intervals present in our data. For the short, medium, and long interval subsets, we conducted the analysis with mixed effects models since these subsets still had multiple visits per participant. For the first and last visit analysis, we used linear models.

**
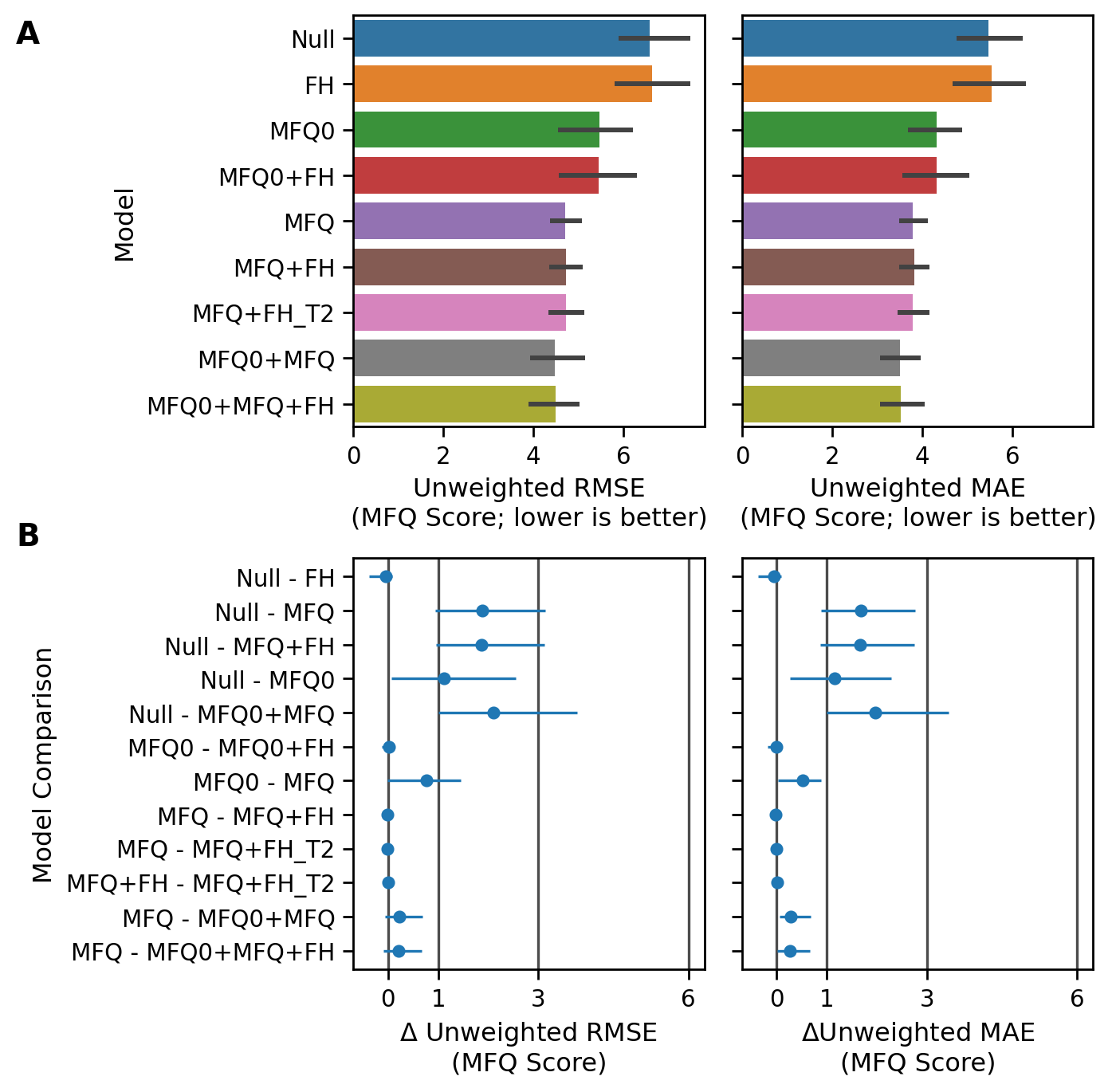
**

**Figure S17:** The top panel (A) shows the unweighted root mean squared error (RMSE) and mean absolute error (MAE) for each model along with bootstrap 99.9% confidence intervals. The lower panel (B) shows comparisons of interest between models in A. Each dot represents the mean difference in RMSE or MAE, while the error bars represent 99.9% confidence intervals. 6 points on the MFQ is the minimum clinical difference, and half of this value is also shown.


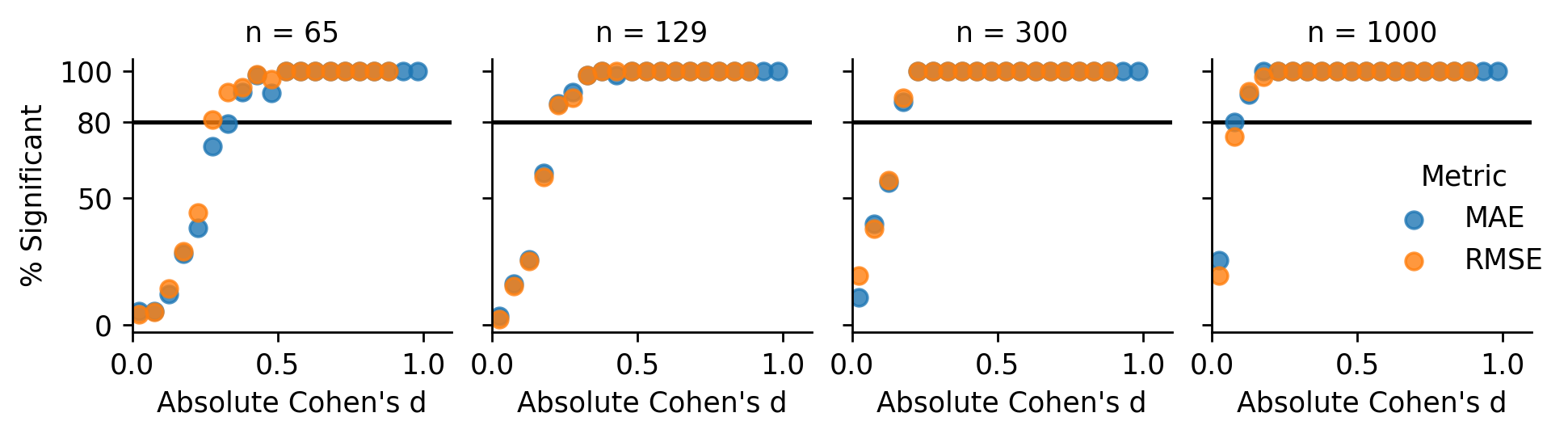


**Figure S18**: Power curves for different sample sizes for the MFQ analysis. % Significant corresponds to the percent of simulations at a given Absolute Population Cohen’s d that found a significant result and is equivalent to the power of the analysis. The horizontal line indicates 80% power. Our analysis had n = 129. Note that these analyses assume the same distribution of number of assessments per participant as we observed in our sample.


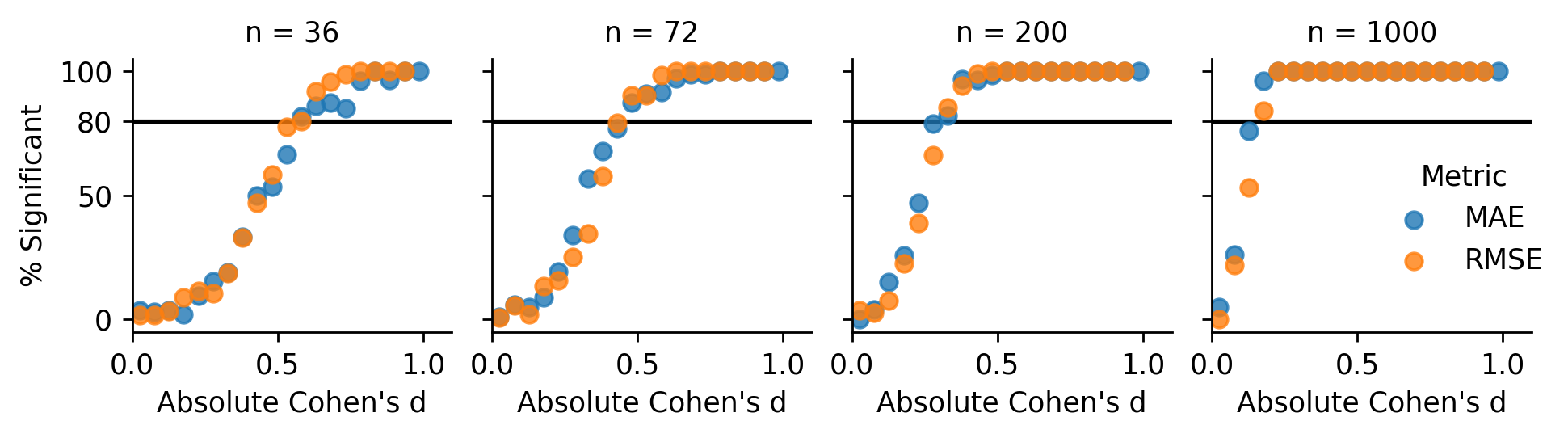


**Figure S19**: Power curves for different sample sizes for the Weeks of Depression analysis. % Significant corresponds to the percent of simulations at a given Absolute Population Cohen’s d that found a significant result and is equivalent to the power of the analysis. The horizontal line indicates 80% power. Our analysis had n = 72.
